# Influence of Pasteurization on Maillard Reaction in Lactose-Free Milk

**DOI:** 10.3390/molecules28207105

**Published:** 2023-10-15

**Authors:** Haixin Bi, Yingbin Wang, Yujuan Guo, Ziyan Liao, Zhiguo Na

**Affiliations:** 1 School of Food Engineering, Harbin University of Commerce, Harbin 150028, China; bihaixin0615@163.com; 2 College of Food Engineering, East University of Heilongjiang, Harbin 150060, China; 15561564371@163.com (Y.W.); 18845556609@163.com (Y.G.); 18804623565@163.com (Z.L.)

**Keywords:** lactose-free milk, Maillard reaction, furosine, 5-hydroxymethylfurfural, color difference, fluorescence intensity, pasteurized

## Abstract

In order to improve the safety and quality of lactose-free milk (LFM) Maillard reaction products (MRPs), this study used raw cow’s milk as raw material and lactase hydrolysis to prepare LFM, which was heat-treated using pasteurization and then placed in storage temperatures of 4 °C, 25 °C and 37 °C to investigate the changes in the Maillard reaction (MR). The results of the orthogonal test showed that the optimal conditions for the hydrolysis of LFM are as follows: the hydrolysis temperature was 38 °C, the addition of lactase was 0.03%, and the hydrolysis time was 2.5 h. Under these conditions, the lactose hydrolysis rate reached 97.08%, and the lactose residue was only 0.15 g/100 g as determined by high-performance liquid chromatography (HPLC), complying with the standard of LFM in GB 28050–2011. The contents of furoamic acid and 5-hydroxymethylfurfural were determined by high-performance liquid chromatography, the color difference was determined by CR-400 color difference meter, and the internal fluorescence spectrum was determined by F-320 fluorescence spectrophotometer. The test results showed that the variation range of furosine in lactose-free milk after pasteurization was 44.56~136.45 mg/100g protein, the range of 5-hydroxymethylfurfural (HMF) was 12.51~16.83 mg/kg, the color difference ranges from 88.11 to 102.53 in L*, from −0.83 to −0.10 in a*, and from 1.88 to 5.47 in b*. The furosine content of LFM during storage at 4, 25, and 37 °C ranged from 44.56 to 167.85, 44.56 to 287.13, and 44.56 to 283.72 mg/100 g protein, respectively. The average daily increase in protein content was 1.18–3.93, 6.46–18.73, and 15.7–37.66 mg/100 g, respectively. The variation range of HMF was 12.51~17.61, 12.51~23.38, and 12.51~21.1 mg/kg, and the average daily increase content was 0.03~0.07, 0.47~0.68, and 0.51~0.97 mg/kg, respectively. During storage at 4 °C, the color difference of LFM ranged from 86.82 to 103.82, a* ranged from −1.17 to −0.04, and b* ranged from 1.47 to 5.70. At 25 °C, color difference L* ranges from 72.09 to 102.35, a* ranges from −1.60 to −0.03, b* ranges from 1.27 to 6.13, and at 37 °C, color difference L* ranges from 58.84 to 102.35, a* ranges from −2.65 to 1.66, and b* ranges from 0.54 to 5.99. The maximum fluorescence intensity (FI) of LFM varies from 131.13 to 173.97, 59.46 to 173.97, and 29.83 to 173.97 at 4, 25, and 37 °C. In order to reduce the effect of the Maillard reaction on LFM, it is recommended to pasteurize it at 70 °C—15 s and drink it as soon as possible during the shelf life within 4 °C.

## 1. Introduction

Cow’s milk is rich in protein, amino acids, fats, lactose, minerals, vitamins, more than 100 kinds of chemical compositions, is called “the closest to the ideal natural food”, and has the physiological functions of promoting the growth and development of infants and young children, anti-cancer properties, and preventing osteoporosis and rickets [1,2,3,4]. Although cow’s milk is rich in nutrients, most infants, adolescents, adults, and the elderly experience gastrointestinal symptoms, such as diarrhea, bloating, cramps, and nausea, about one hour after consuming cow’s milk, which is known as “lactose intolerance” [5]. The prevalence of lactose intolerance is high in Asia and Oceania, with prevalence rates of more than 90% in Korea, Vietnam, Myanmar, and the Solomon Islands [6]. Lactase, also known as *β*-galactosidase (EC.3.2.1.23), has the function of hydrolyzing lactose, which is found in animals, plants, and microorganisms. The obstacle to the presence of lactose in dairy products can be overcome by hydrolysis of lactose by the addition of lactase to cow’s milk, which is currently the most recognized and safest method [7,8,9,10].

Lactose is hydrolyzed by lactase to produce glucose and galactose, in which the carbonyl group can condense with the amino group in lysine to produce elanoidin, which results in the Maillard reaction (MR), affecting the nutritional value, safety, and quality of cow’s milk [11]. Furosine is a kind of Amadori compound formed by the reaction of reducing sugar with an amino group and then hydrolyzed by acid to form the stabilized product of the primary stage of Maillard, and its content will increase with the enhancement of the heat treatment intensity, so it can indirectly reflect the reaction process of the primary stage of Maillard, and it is one of the main indexes for evaluating the intensity of heat treatment of cow’s milk [12]. 5-Hydroxymethylfurfural (HMF) is one of the representative products of the middle stage of the MR, which is metabolized in the human body to produce derivatives that are carcinogenic, and its production is not only proportional to the degree of browning of cow’s milk but also correlates with the intensity of heat treatment of cow’s milk [13,14,15]. The color of milk can indicate the degree of the MR, and as its reaction process accelerates, the melanoidins generated at the end stage affect the color of the food, which, to a certain extent, influences the sensory value of the food and the consumer’s desire to buy it [16]. Small molecules produced by degradation in the terminal phase of the MR are precursors of brown substances and have fluorescent properties. The fluorescence intensity (FI), which is related to its state of production, consumption, type of reactant, and degree of reaction [17,18].

In 1862, French biologists invented pasteurization technology, which is a method to kill most of the pathogens and spoilage bacteria through a low temperature treatment of raw cow’s milk, which not only maximizes the retention of high-quality proteins and other nutrients in raw cow’s milk, but also effectively kills pathogenic microorganisms in it; thus, the heat sterilization process of pasteurized milk is crucial [19]. The research shows that pasteurization heat treatment can affect the Maillard reaction of milk, Lambros et al. heat-treated raw cow’s milk under pasteurization conditions at 80 °C and 4 s, the furosine content was measured to be 8.9 mg/100 g protein and the *β*-lactoglobulin content was 2716 mg/L [20]. In addition, different storage temperatures of lactose-hydrolyzed milk can also affect the MR, Zhang et al., compared the HMF and color changes in UHT pure milk and UHT lactose-hydrolyzed milk stored at 20, 30, and 40 °C for 1 year, and the results showed that the HMF content of UHT lactose-hydrolyzed milk increased with the rise of storage temperature and the extension of storage time as compared with that of UHT pure milk; the color of UHT lactose-hydrolyzed milk changed (∆E > 10) after 4 months of storage at 30, 40 °C [21].

Therefore, this study was conducted to determine the MRPs (furosine, HMF) of LFM after pasteurization and storage, and to analyze the color (L*, a*, b*).

## 2. Results and Discussion

### 2.1. Single-Factor Experiment on the Preparation of LFM by Lactase Hydrolysis

**Figure 1:**
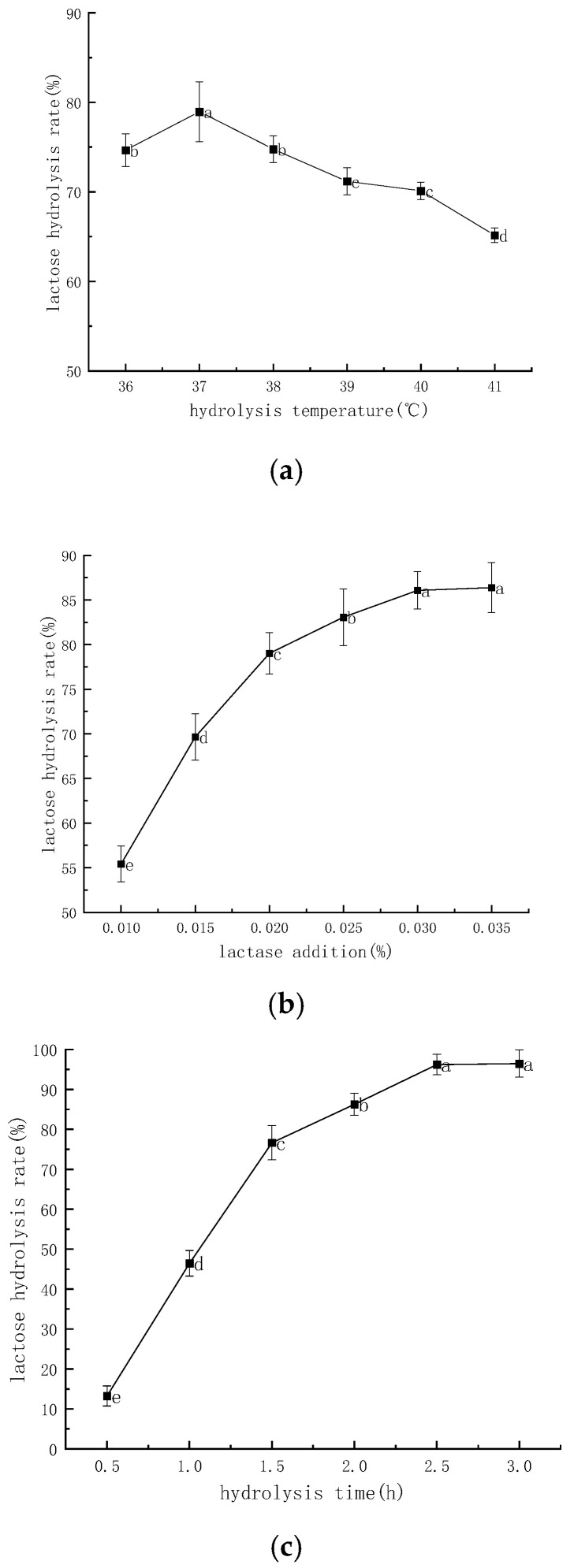
Effect of different factors on the rate of lactose hydrolysis. (**a**) Effect of hydrolysis temperature on the hydrolysis rate of lactose. (**b**) Effect of lactase addition on the rate of lactose hydrolysis. (**c**) Effect of hydrolysis time on the rate of lactose hydrolysis. Note: different lowercase letters indicate significant differences (*p* < 0.05).

**Figure 2:**
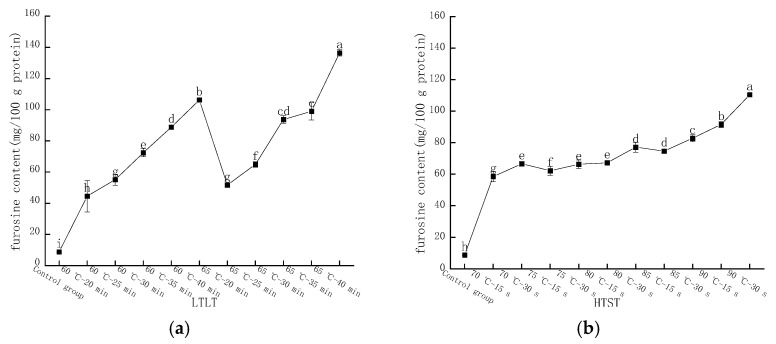
Changes in furosine content of LFM after pasteurization. (**a**) Low temperature, long time pasteurization. (**b**) High temperature, short time pasteurization. Different lowercase letters indicate significant differences (*p* < 0.05).

Figure 1a–c shows the effect of hydrolysis temperature, lactase addition, and hydrolysis time on lactose hydrolysis rate, respectively. As shown in Figure 1a, the lactose hydrolysis rate was the highest at the hydrolysis temperature of 37 °C, which was 78.97%; the lactose hydrolysis rate decreased to 65.17% at 41 °C, which showed that the lactase activity was greatly affected by temperature. The hydrolysis rate increased significantly (*p* < 0.05) when the hydrolysis temperature was lower than 37 °C. When the hydrolysis temperature exceeded 37 °C, the hydrolysis rate of lactose decreased significantly with the increase in hydrolysis temperature (*p* < 0.05), which may be due to the gradual inactivation of lactase with the increase in hydrolysis temperature, resulting in the decrease in lactose hydrolysis rate [22]. It can be concluded that 37 °C is the optimum temperature for the hydrolysis of the lactase. Figure 2b shows the effect of lactase addition on lactose hydrolysis rate. The lactose hydrolysis rate increased significantly (*p* < 0.05) when the lactase addition was less than 0.03%. The lactose hydrolysis rate reached its maximum value of 86.39% when the lactase addition was 0.035%, which may be due to the increased probability of lactase–lactose binding when the lactase addition was increased [23]. It can be seen that increasing the amount of lactase addition favors the hydrolysis of lactose. And the results at 0.035% of lactase addition were not significantly different from the results at 0.03% (86.08%) (*p* > 0.05); considering the cost of experimental and production of practical applications; thus, it was determined that the amount of lactase added was 0.03%.

Figure 1c shows the effect of hydrolysis time on lactose hydrolysis rate. The lactose hydrolysis rate increased significantly (*p* < 0.05) at a hydrolysis time of less than 2.5 h. It may be that prolonging the hydrolysis time facilitates the binding of lactase to lactose [24] and, therefore, lactose hydrolysis as well. The hydrolysis rate was only 13.24% when the hydrolysis time was 0.5 h, while the hydrolysis rate reached the maximum value of 96.43% when the hydrolysis time was 3 h. This result was not significantly different from that of 2.5 h (96.21%) (*p* > 0.05), and the hydrolysis time of 2.5 h was determined, taking into consideration the efficiency of the experiments and the actual time cost of the production. This trend was also confirmed in the study by Sining Li et al. and suggests that cow’s milk at 2.5 h and later meets the needs of lactose intolerant patients [25].

### 2.2. Orthogonal Test for the Preparation of LFM by Lactase Hydrolysis

Based on the results of single-factor experiments, it was found that all three factors, lactose hydrolysis temperature, lactase addition, and hydrolysis time, had a significant effect on the lactose hydrolysis rate, and Table 1 shows the results of orthogonal experiments on lactase hydrolysis for the preparation of LFM.

The extreme difference value-R can reflect the order of the influence of each factor on the lactose hydrolysis rate as follows: hydrolysis temperature > lactase addition > hydrolysis time; according to the value of k, it can be obtained that the lactose hydrolysis rate increases with the increase in the values of the above three factors; the optimal hydrolysis process was A_3_B_3_C_3_, i.e., the hydrolysis temperature was 38 °C, the addition of lactase was 0.03%, and the hydrolysis time was 2.5 h, and the lactose hydrolysis rate was 97.08%, which was significantly higher than that of the results in Table 1 (92.64%), which proved that the process parameters were reasonable. According to the relevant provisions of the *General Principles for Nutrition Labeling of Prepackaged Foods* issued by the Ministry of Health of the People’s Republic of China (GB 28050–2011), lactose content of less than 0.5 g/100 g is regarded as LFM. According to the relevant provisions of Appendix A of the General Principles for Nutrition Labeling of Prepackaged Foods issued by the Ministry of Health of the People’s Republic of China (GB 28050–2011), lactose content of less than 0.5 g/100 g is regarded as lactose-free cow’s milk. After the optimization of the orthogonal hydrolysis test, the lactose residue in milk is only 0.15 g/100 g, which meets the requirement of LFM.

### 2.3. Changes in Furosine Content

#### 2.3.1. Changes in Furosine Content after Pasteurization

The furosine content in LFM was determined by HPLC with a peak time around 6.2 min. Figure 2a shows the changes in furosine content in LFM after low temperature, long time pasteurization (LTLT), and the results were significantly different. At 60 °C and a heat pasteurization time of 20, 25, 30, 35, and 40 min, the furosine content varied in the range of 44.56–106.28 mg/100 g protein, while the furosine content of LFM samples varied in the range of 51.69–136.45 mg/100 g protein when the heat pasteurization time was kept constant, and the temperature reached 65 °C. It was shown that the furosine content gradually increased as the LTLT temperature of LFM was increased. Furosine is an amino acid produced by hydrolysis of Amadori acid at the primary stage of the MR, in which about 20%~52% of the product will form furosine, which is one of the indicators for evaluating the degree of heat exposure, the degree of the MR and the loss of lysine, and its content is related to the pasteurization temperature and time, and the higher the pasteurization temperature and the longer the time, the higher the furosine content [26].

Figure 2b shows the furosine content in LFM after high temperature, short time pasteurization (HTST). At the pasteurization conditions of 70, 75, 80, 85, and 90 °C for 15 s, the furosine content varied from 58.52 to 91.60 mg/100 g protein; when the time reached 30 s and the temperature was constant, the furosine contents of LFM samples were 62.59, 66.3, 77.13, 82.80, and 110.42 mg/ 100 g protein.

In conclusion, the furosine content of LFM after both LTLT and HTST was elevated with the prolongation of the pasteurization time and the increase in the temperature, which was in agreement with the findings of Xinxin Wei [27].

#### 2.3.2. Changes in Furosine Content during Storage at 4 °C

**Figure 3:**
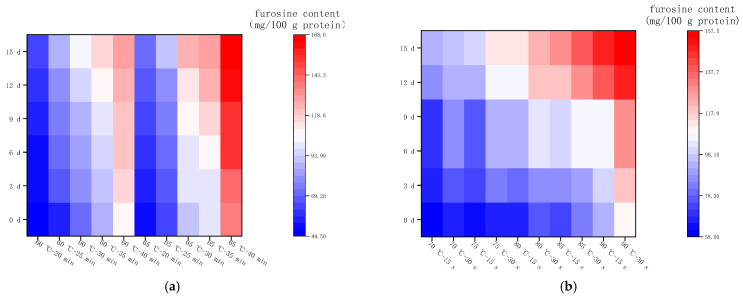
Changes in furosine content in LFM during storage at 4 °C after pasteurization. (**a**) Low temperature, long time pasteurization. (**b**) High temperature, short time pasteurization.

Figure 3a shows the changes in furosine content in LFM after LTLT during the storage (4 °C), which ranged from 44.56 to 167.85 mg/100 g protein. When the pasteurization temperature was 60 °C and the time was 20, 25, 30, 35, and 40 min, the daily average increase in furosine content was 1.16, 2.08, 2.00, 1.79, and 1.64 mg/100 g protein, respectively; furthermore, when the time was constant and the temperature was increased to 65 °C, the content increased by a daily average of 1.18, 1.72, 1.97, 2.06, and 2.09 mg/100 g protein. This indicates that furosine content increased the least daily under the condition of 60 °C−20 min, and the daily increase in its content was the most at 90 °C−30 s.

Figure 3b shows the changes in furosine content in LFM after HTST during the storage (4 °C). The variations ranged from 58.52 to 157.24 mg/100 g protein, and the daily increase in furosine content was 2.05–3.93 mg/100 g protein, which was slightly higher than the results of the samples sterilized at low temperature for a long time. In summary, the furosine content of LFM increased slowly during the storage period at 4 °C after pasteurization, and the initial degree of the MR was mild. This is consistent with the findings of Xinyu Hao et al. [28].

#### 2.3.3. Changes in Furosine Content during Storage at 25 °C

**Figure 4:**
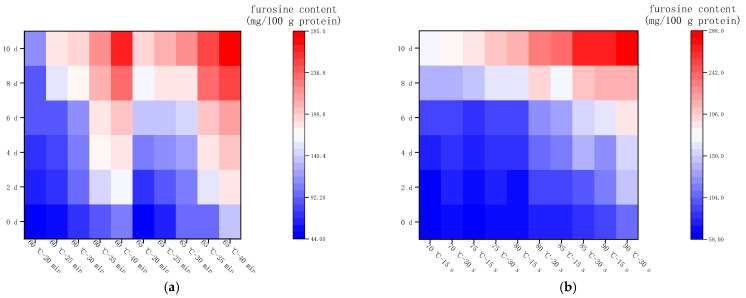
Changes in furosine content in LFM during storage at 25 °C after pasteurization. (**a**) Low temperature, long time pasteurization. (**b**) High temperature, short time pasteurization.

Figure 4a shows the changes in furosine content in LFM after LTLT during the storage (25 °C); under this condition, the content varied from 44.56 to 284.44 mg/100 g protein, with an average daily increase of 6.46–15.58 mg/100 g protein. Figure 4b shows the changes in furosine content in LFM after HTST during the storage (25 °C). The variations ranged from 58.52 to 287.13 mg/100 g protein, with an average daily increase of 11.27–18.73 mg/100 g protein. In conclusion, the furosine content of LFM increased rapidly during the storage (25 °C) after both LTLT and HTST treatments, while the LFM treated with HTST had a higher average daily furosine production and a faster initial degree of MR. This is consistent with the results of Sunds et al., who determined the furosemide content in UHT cow’s milk during storage at 20 °C, which showed an increasing trend with the storage time [29].

#### 2.3.4. Changes in Furosine Content during Storage at 37 °C

**Figure 5:**
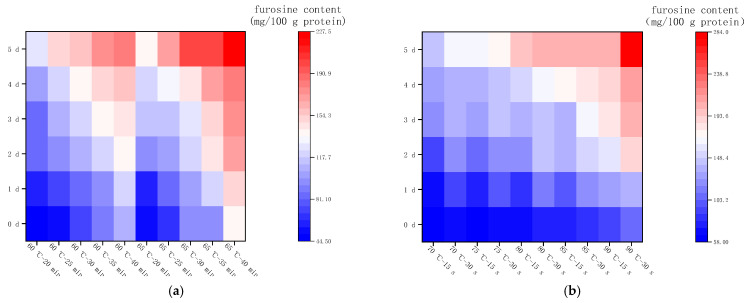
Changes in furosine content in LFM during storage at 37 °C after pasteurization. (**a**) Low temperature, long time pasteurization. (**b**) High temperature, short time pasteurization.

Figure 5a shows the changes in furosine content in LFM after LTLT during the storage (37 °C). The furosine content of LFM samples varied from 44.56 to 227.45 mg/100 g protein, with an average daily variation of 15.70–20.83 mg/100 g protein. Figure 5b shows the changes in furosine content in LFM after HTST during the storage (37 °C). The content varied from 58.52 to 283.72 mg/100 g protein, with a daily increase of 17.93–37.66 mg/100 g protein, with the slowest growth rate of furosine content in the samples thermally sterilized at 70 °C—15 s, and the fastest growth in the samples sterilized at 90 °C—30 s. Therefore, the furosine content of LFM after pasteurization increased rapidly during the storage (37 °C), and the initial degree of its MR was drastic. This is close to the results of Troise et al. for commercially available LFM stored at 37 °C, which showed the lowest range of change in furosine content from 0 to 9 d to 208.9 mg/100 g protein [30]. In a previous study [31], the furosine production in UHT milk stored at 4, 25, and 37 °C, it was shown that the slowest production rate was found at 4 °C, the fastest at 25 °C and the fastest at 37 °C, which is consistent with the results of the present study.

### 2.4. Changes in the Content of HMF

#### 2.4.1. Changes in HMF Content after Pasteurization

**Figure 6:**
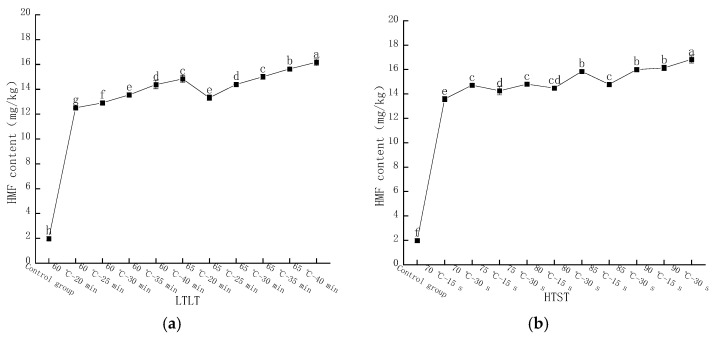
Changes in HMF content after pasteurization. (**a**) Low temperature, long time pasteurization. (**b**) High temperature, short time pasteurization. Different lowercase letters indicate significant differences (*p* < 0.05).

The content of HMF in LFM was determined by HPLC, and the peak time was around 3.8 min. As shown in Figure 6a, the HMF content in LFM after LTLT showed different increasing trends. The content of HMF varied in the range of 12.51–14.84 mg/kg at thermal pasteurization of 60 °C for 20, 25, 30, 35, and 40 min, respectively; furthermore, when the pasteurization time remained unchanged and the temperature reached 65 °C, the content ranged from 13.32 to 16.18 mg/kg, and the reason for this phenomenon might be that with the increase in pasteurization intensity, the lysine in the samples participated in the MR and accelerated the reaction process [32]. As shown in Figure 6b, the HMF content in the samples was 13.58, 14.26, 14.48, 14.78, and 16.13 mg/kg at a pasteurization time of 15 s and temperatures of 70, 75, 80, 85, and 90 °C, respectively; and the HMF content was increased to 14.72, 14.8, and 15.84 when the pasteurization time was prolonged to 30 s, the HFM content was increased to 14.72, 14.8, 15.84, 16, and 16.83 mg/kg, respectively.

HMF, as one of the representative furfural organic compounds in the middle stage of the MR, is not usually present in raw cow’s milk, and its content is related to the intensity of heat treatment and storage temperature, which is one of the substances for evaluating the degree of MR [33,34]. The above study showed that the content of HMF in LFM after HTST was slightly higher than the result of LTLT, which was consistent with the findings of Meng Gao et al. [35].

#### 2.4.2. Changes in HMF Content during Storage at 4 °C

**Figure 7:**
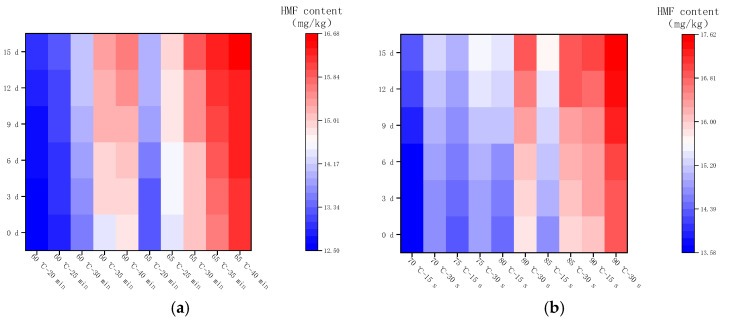
Changes in HMF content in LFM during storage at 4 °C after pasteurization. (**a**) Low temperature, long time pasteurization. (**b**) High temperature, short time pasteurization.

Figure 7 shows the changes in HMF in LFM after pasteurization during storage (4 °C). The content of HMF ranged from 12.51 to 16.67 mg/kg after LTLT, with an average daily increase in the range of 0.03–0.07 mg/kg, whereas the content of HMF after HTST was in the range of 13.58–17.61 mg/kg, with an average daily increase in the range of 0.03–0.07 mg/kg. It was found that the HMF content of LFM after HTST was slightly higher than that of LTLT during the storage (4 °C), and the average daily increase was less, which may be related to the stability of LFM and the inter-conversion of the intermediate products of the MR. This result is close to the range of HMF in brown yogurt (9.87–26.67 mg/kg) during storage at 4 °C by Risiu Bi et al. [36].

#### 2.4.3. Changes in HMF Content during Storage at 25 °C

**Figure 8:**
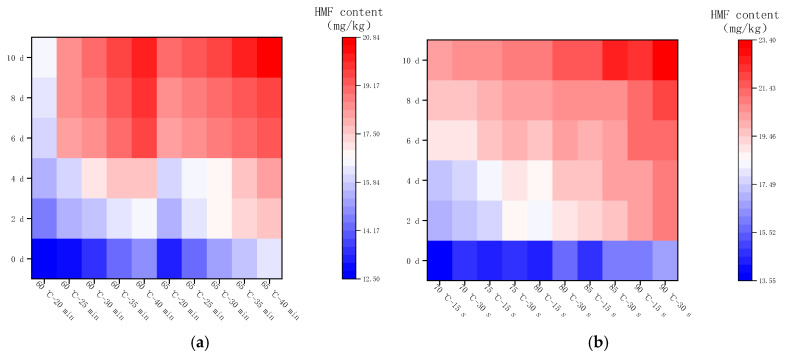
Changes in HMF content in LFM during storage at 25 °C after pasteurization. (**a**) Low temperature, long time pasteurization. (**b**) High temperature, short time pasteurization.

Figure 8 shows the changes in HMF content in LFM after pasteurization during the storage (25 °C), in which the range of HMF content was 12.51–20.83 mg/kg after LTLT, with an average increase of 0.47–0.57 mg/kg per day, and the range of HMF content was 13.58–23.38 mg/kg after HTST, with an average increase of 0.58–0.68 mg/kg per day. Therefore, the content of HMF and its average daily growth were slightly higher in LFM sterilized by HTST compared with LTLT. This finding is consistent with the results reported by Xiaodi Jia et al. for raw cow’s milk heat-treated by pasteurization and ultra-high-temperature pasteurization, who explored the changes in HMF content during storage (25 °C), which gradually accumulated [37].

#### 2.4.4. Changes in HMF Content during Storage at 37 °C

**Figure 9:**
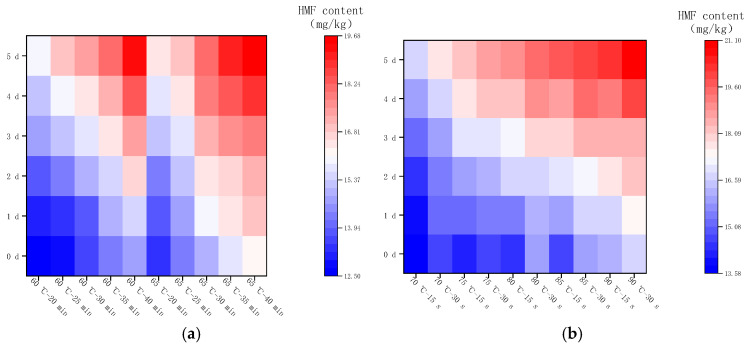
Changes in HMF content in LFM during storage at 37 °C after pasteurization. (**a**) Low temperature, long time pasteurization. (**b**) High temperature, short time pasteurization.

Figure 9 shows the changes in HMF content in LFM after pasteurization during the storage (37 °C). The HMF content varied from 12.51 to 19.67 mg/kg with an average daily increase of 0.51–0.88 mg/kg after LTLT, while it varied from 13.58 to 21.1 mg/kg with an average daily increase of 0.61–0.97 mg/kg after HTST. It can be concluded that the HMF content and its average daily growth content were slightly higher after HTST compared with the results of LTLT under the same storage conditions. Similar studies have been reported [38], in which they used the UHT method to heat treat raw cow’s milk to investigate the generation of HMF during the shelf-life, and the results showed that the HMF content increased rapidly.

### 2.5. Color Analysis

#### 2.5.1. Changes in Color after Pasteurization

The color change in LFM after pasteurization is shown in Table A1. The accumulation of substances, such as melanoidins produced at the end of the MR, affects the color of the food, and the changes in their color parameters reflect the degree of the MR.

Compared with the control group (LFM without heat treatment), L*, a*, and b* of LFM were significantly decreased (*p* < 0.05) after pasteurization, indicating that the change in the Maillard color of buttermilk was related to the time and temperature of heat treatment. Among them, L* value varied from 88.11 to 102.35, with an overall decrease of 4.69–17.95% (*p* < 0.05), and L* value first decreased and then increased after LTLT(60 °C–20 min, 60 °C–25 min, 60 °C–30 min, 60 °C–35 min, and 60 °C–40 min), however, there was no significant change in the rest of the pasteurization conditions. It indicated that the LFM darkens after pasteurization, which might be caused by the accumulation of brown pigments, such as melanoidins produced at the end of the MR [39]. The a* value varied from −0.83 to −0.10 and moved towards negative values, which indicated that there was a tendency for LFM to turn slightly green after pasteurization, which is in agreement with the findings of Yuecheng Meng et al. [40]. The b* values varied from 1.88 to 5.47, with an overall decrease of 36.54–78.19%, indicating that LFM tends to be yellow after pasteurization.

#### 2.5.2. Color Change during Storage at 4 °C

The color changes in LFM after pasteurization during the storage (4 °C) are presented in Table A2. The L* values ranged from 86.82 to 103.82; a* values ranged from −1.17 to −0.04, both negative; and b* values varied in the range of 1.47–5.70. Compared with the control group (unpasteurized cow’s milk), the L* values decreased by 3.32–19.15%, but at a slower rate; the b* values decreased by 33.87–82.95%. This shows that LFM darkens and tends to be green during the storage (4 °C), which is consistent with the findings of Xiaoqian Yang et al. [41].

#### 2.5.3. Color Change during Storage at 25 °C

The color changes in LFM after pasteurization during the storage (25 °C) is shown in Table A3. The L* values varied from 72.09 to 102.35, which decreased from 4.69% to 32.87% compared with the control group, proving that the storage temperature plays a certain role in the color of LFM, and the brown substances such as melanoidins were generated, which promoted the MR; the a* values varied from −1.60 to −0.03, which were all negative and tended to turn green; and the b* values varied from 1.27 to 6.13, all positive values, and the color tends to be yellow. In the study on the effect of storage temperature on the MR of UHT buttermilk, it was stored at 20 °C and above and the color indexes were measured periodically, and the results obtained were consistent with the present study [42], with a decreasing trend in L*, a* being negative and tending to be green, and b* being positive and tending to be yellow.

#### 2.5.4. Color Change during Storage at 37 °C

Table A4 shows the color changes in LFM after pasteurization during the storage (35 °C). L* varied from 58.84 to 102.35, with a decrease of 4.69–45.21% compared with the control group, and the L* of some samples was lower than that of the storage (4 °C and 25 °C), which indicated that the color of LFM was darker during the storage (37 °C), and the compounds of furosine, HMF, and furfural continued to react to produce more brown pigments to accelerate the MR process; moreover, the a* varied from −2.65 to 1.66, with some samples tending to be red, and the b* varied from 0.54 to 5.99, all of which were positive, but b* value of the samples treated with 60–30 stored at 37 °C for 1 d were lower than those of the storage (4 °C and 25 °C), and the reasons for this need to be further investigated.

This result is consistent with the results reported by Ji-En Tan et al. [43] on the change in color difference values of the simulated systems of ovalbumin and glucose MR at 4, 25, and 37 °C, who concluded that L* was the lowest at 37 °C, intermediate at 25 °C, and the largest at 4 °C; a* was negative at both 4 °C and 25 °C; and b* was positive at 4, 25, and 37 °C. In the study by Shengnan Yi et al. [44], raw cow’s milk was subjected to ultra-pasteurization treatment using 120 °C and 15 s. The samples were stored at 4, 25, and 37 °C, and the color changes were determined periodically, and the results showed a significant decrease in L* at 25 and 37 °C, and changes in a* and b*, which are consistent with the results of the present study.

### 2.6. Endogenous Fluorescence Spectroscopy (EFS) Analysis

#### 2.6.1. EFS after Pasteurization

**Figure 10:**
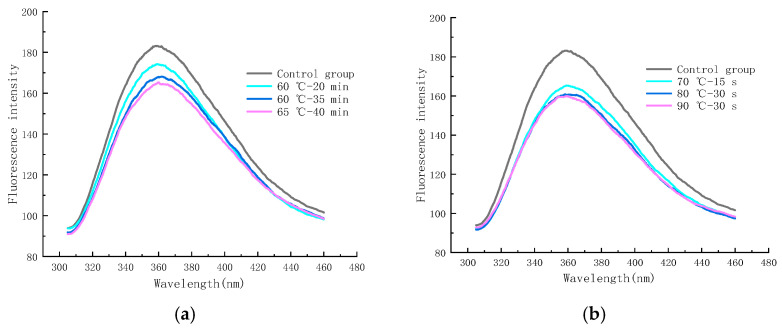
Trends in endogenous fluorescence spectra of LFM after pasteurization. (**a**) Low temperature, long time pasteurization. (**b**) High temperature, short time pasteurization.

As shown in Figure 10a, the final stage of the MR can produce a substance with fluorescence, which can react with neighboring proteins or amino acids and is a precursor of brown substances, and its FI can be used to evaluate the degree of the MR and the state of production of small molecules [45,46]. The maximum FI of the control group was 188.16 (at an emission wavelength of 358.4 nm), whereas the samples sterilized under 60 °C–20 min, 60 °C–35 min, and 65 °C–40 min conditions showed a maximum FI of 173.97 (360.8 nm), 167.95 (361 nm), and 164.41 (361.2 nm), respectively. It can be seen that the maximum FI of LFM shifted to the long-wave direction after LTLT, and the FI decreased significantly with the increase in the pasteurization intensity as compared to the control group, which may be due to the fact that the hydrolyzed glucose and galactose combined with partially unhydrolyzed lactose and protein shielded the tryptophan, exposing the tryptophan residues to the hydrophilic environment [47,48], which indicated that some small molecules of fluorescence substances were generated at the end of the MR and that the LTLT facilitated the MR.

As shown in Figure 10b, the maximum FI of LFM reached 165.37 (359 nm), 160.81 (360.8 nm), and 159.97 (359.4 nm) after pasteurization at 70 °C—15 s, 80 °C—30 s, and 90 °C—30 s conditions, respectively. The FI of LFM after HTST was lower than that of the control group, and the FI decreased with the increase in the pasteurization intensity, and the wavelengths of its maximum FI ranged from 359 to 360.8 nm; therefore, the HTST is beneficial to the MR, which is consistent with the results of the LTLT. The above-mentioned results showed that the variation in the maximum FI of LFM after pasteurization ranged from 159.97 to 173.97, which was slightly lower than that of the control group, indicating that pasteurization accelerated the degree of the MR.

This is consistent with the results of studies on the effect of the MR of soybean isolate proteins and fructose under ultra-high-pressure conditions, in which Zihuan Wang et al. concluded that the maximum wavelength of the MR products shifted to the longer wavelengths (redshift) and that the maximum FI decreased [49].

#### 2.6.2. EFS during Storage at 4 °C

**Figure 11:**
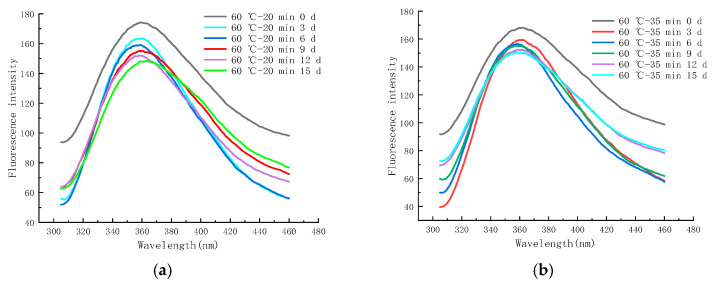

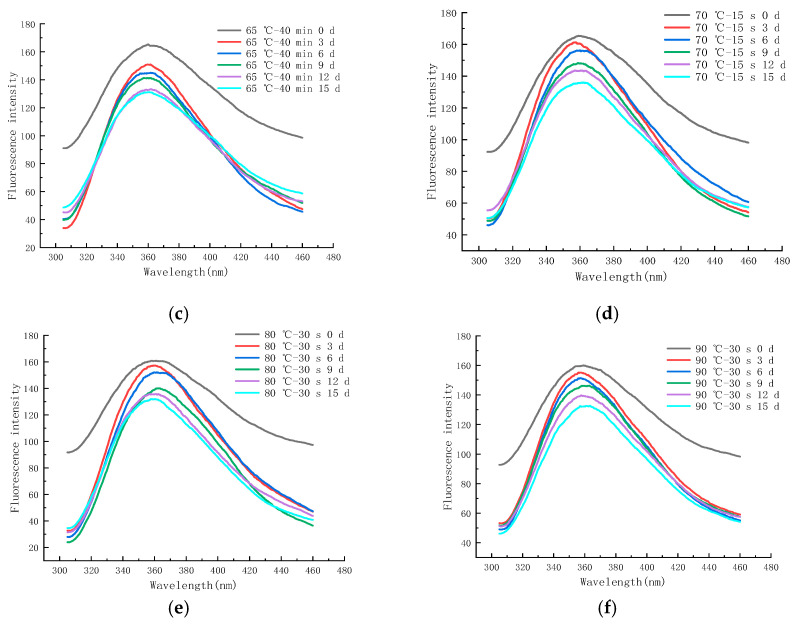
Trends of EFS of LFM during storage at 4 °C after pasteurization. (**a**) 60 °C–20 min. (**b**) 60 °C–35 min. (**c**) 65 °C–40 min. (**d**) 70 °C–15 s. (**e**) 80 °C–30 s. (**f**) 90 °C–30 s.

Figure 11a–f shows the endogenous fluorescence trends of LFM treated with different pasteurization conditions (60 °C—20 min, 60 °C—35 min, 65 °C—40 min, 70 °C—15 s, 80 °C—30 s, and 90 °C—30 s) during the storage (4 °C). From the figure, it was found that the maximum FI of LFM after pasteurization (60 °C—20 min) ranged from 148.52 to 173.97 (Figure 11a), whereas for the same storage conditions, the maximum FI of LFM pasteurized by 65 °C–40 min ranged from 131.13 to 164.41 (Figure 11c). This indicates that the maximum FI of LFM after different pasteurization treatments ranged from 131.13 to 173.97 during the storage (4 °C), which is a small change so that 4 °C has a small effect on the MR of LFM. The maximum FI decreased with longer storage time, which may be due to the shielding effect on tryptophan with the accumulation of the products of the MR [50].

#### 2.6.3. EFS during Storage at 25 °C

**Figure 12:**
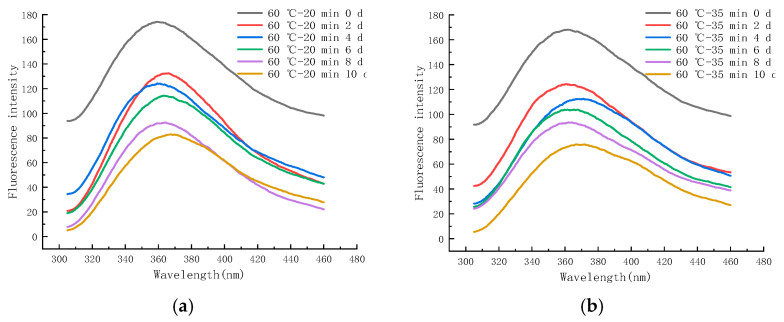

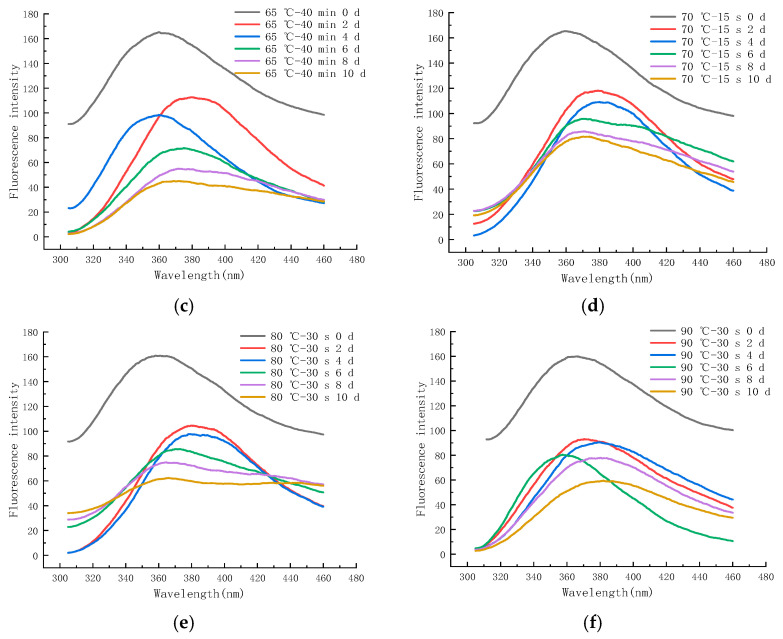
Trends of EFS of LFM during storage at 25 °C after pasteurization. (**a**) 60 °C–20 min. (**b**) 60 °C–35 min. (**c**) 65 °C–40 min. (**d**) 70 °C–15 s. (**e**) 80 °C–30 s. (**f**) 90 °C–30 s.

Figure 12a–f shows the endogenous fluorescence trends of LFM treated with different pasteurization conditions (60 °C–20 min, 60 °C–35 min, 65 °C–40 min, 70 °C–15 s, 80 °C–30 s, and 90 °C–30 s) during the storage (25 °C). The maximum FI of LFM after 60–20 pasteurization ranged from 82.93 to 173.97 (Figure 12a), and the maximum FI of LFM after 65–40 pasteurization ranged from 45.12 to 164.41 (Figure 12c). From the FI trends of all the samples during the storage, the maximum FI ranged from 59.46 to 173.97, which was lower than that of the 4 °C, and the intensity varied greatly with the extension of time, which indicated that LFM had a more intense MR during the storage (25 °C), which further contributed to the extent of the MR. Similar results have also been reported [51], Yuanyuan Zhang et al. analyzed the maximum FI of EFS in the simulated systems of sodium caseinate and oat *β*-glucan meridian reaction and noted that the maximum FI of sodium caseinate and oat *β*-glucan polymer system was more variable compared to the control.

#### 2.6.4. EFS during Storage at 37 °C

**Figure 13:**
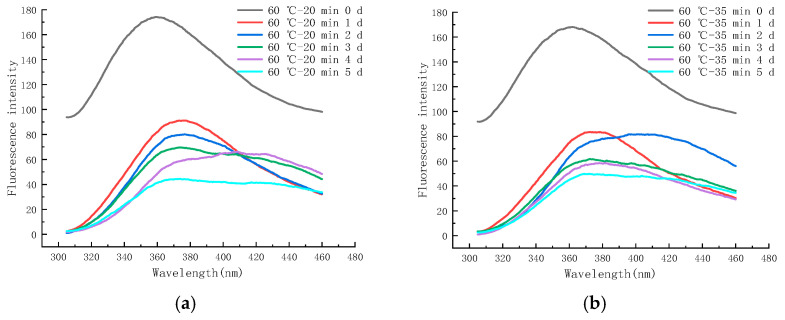

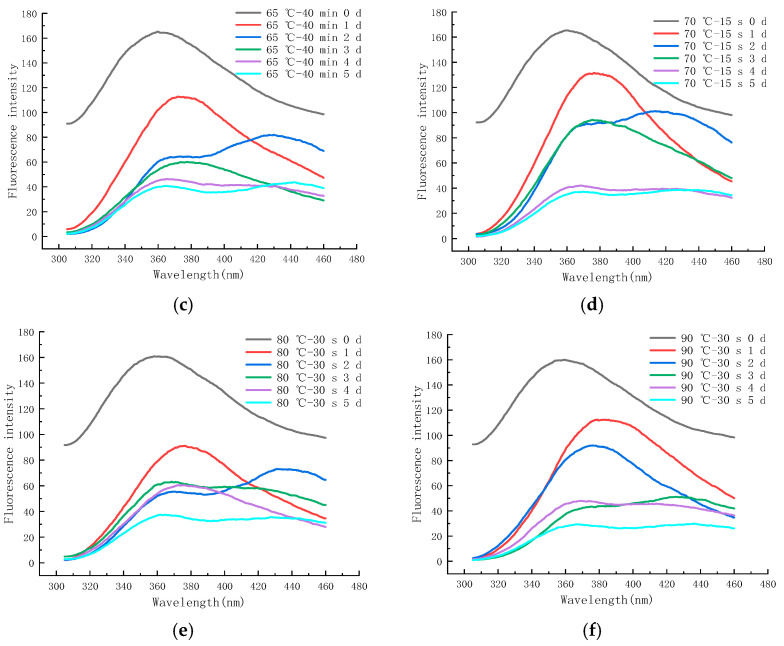
Trends of EFS of LFM during storage at 37 °C after pasteurization. (**a**) 60 °C–20 min. (**b**) 60 °C–35 min. (**c**) 65 °C–40 min. (**d**) 70 °C–15 s. (**e**) 80 °C–30 s. (**f**) 90 °C–30 s.

Figure 13a–f shows the endogenous fluorescence trends of LFM treated with different pasteurization conditions (60 °C–20 min, 60 °C–35 min, 65 °C–40 min, 70 °C–15 s, 80 °C–30 s, and 90 °C–30 s) during the storage (37 °C). The maximum FI of LFM after 60–20 pasteurization was in the range of 44.43~173.97, and after 90–30 pasteurization, the maximum FI of LFM increased from 29.83 to 159.97.

Therefore, according to the FI trends of the samples treated with different pasteurization conditions, it can be concluded that the maximum FI range of LFM was 29.83~173.97, and this result was lower than that of the storage period (between 4 °C and 25 °C), and the range of change was the largest with the extension of storage time. It indicates that LFM has the most intense MR and generates the most fluorescent substances at 37 °C, because the spatial structure of proteins changes as the storage temperature increases, resulting in the decrease in tryptophan content and the increase in hydrophilicity, and the fluorescence of the MR in LFM is burst out [52].

## 3. Materials and Methods

### 3.1. Materials and Instruments

*β*-galactosidase (enzyme activity 5000 NLU/mL) was purchased from Royal DSM Group (Royal DSM of the Netherlands, Heerlen, The Netherlands); lactose, potassium ferricyanide, zinc acetate, sodium hydroxide, 1-phenyl−3-methyl−5-pyrazolone, hydrochloric acid, oxalic acid, trichloromethane, ammonium acetic acid, glacial acetic acid, copper sulphate, and potassium sulphate (the above reagents are pure for analysis) were purchased from Kommeo Chemical Reagents Ltd. (Tianjin, China); methanol and acetonitrile (chromatographic purity) were purchased from Komeo Chemical Reagent Co. Ltd. In total, 100 μg/mL furosine standard (chromatographic purity) was supplied by Alta Science and Technology Co. Ltd. HMF standard (purity: 97%), trifluoroacetic acid (chromatographic purity) was supplied by McLean Bio-chemistry and Technology Ltd. (Shanghai, China).

### 3.2. Experimental Methods

#### 3.2.1. Singe-Factor Experiment on the Preparation of LFM by Lactase Hydrolysis

Raw cow’s milk (500 g) was preheated in a hot water bath (37 °C) for 10 min, and 0.02% lactase was added, and the hydrolysis time was 2 h. During the hydrolysis process, a glass rod was used to stir for 1 min every 10 min to ensure that the lactose was fully hydrolyzed. At the end of hydrolysis, the buttermilk was inactivated in a water bath (70 °C) for 15 s, and then quickly cooled in an ice water bath. During this period, the optimal hydrolysis conditions were determined by calculating the lactose hydrolysis rate by varying the hydrolysis temperature (36, 37, 38, 39, 40, and 41 °C), the amount of lactase added (0.01%, 0.015%, 0.02%, 0.025%, 0.03%, and 0.035%), and the time of hydrolysis (0.5, 1.0, 1.5, 2.0, 2.5, and 3 h), respectively. The optimal hydrolysis conditions were determined by calculating the lactose hydrolysis rate, and all the experiments were measured three times in parallel.

#### 3.2.2. Orthogonal Test for the Preparation of LFM by Lactase Hydrolysis

Based on the singe-factor test, three factors, A (hydrolysis temperature), B (lactase addition), and C (hydrolysis time), were selected to optimize the lactose hydrolysis conditions using the orthogonal test of L_9_ (3^4^) with the lactose hydrolysis rate as the evaluation index. Table 2 shows the orthogonal factor level table.

#### 3.2.3. Lactose Analysis

Lactose was determined as previously described [53], with some modifications. After hydrolysis and enzyme inactivation (70 °C—30 s), the buttermilk sample (2 g) was mixed with water (10 g), and then 1 mL of potassium ferricyanide solution (106 g/L) and 1 mL of zinc acetate solution (220 g/L) were added after dissolution, and then water was added to the mass of solution of 50 g. The sample was mixed well and allowed to stand for 30 min and then filtered through medium-speed filter paper, and then the filtrate was collected and filtered through 0.45 µm organic filter membrane to obtain the buttermilk sample (stored at 4 °C). The filtrate was collected and filtered through 0.45 µm organic filter membrane to obtain the buttermilk sample (stored at 4 °C).

Pipetted 1 mL of lactose standard solution and 1 mL of cow’s milk samples, added 200 µL of sodium hydroxide solution (0.3 mol/L) and 200 µL of 1-phenyl−3-methyl−5-pyrazolone-methanol solution (0.5 mol/L), after mixing with SI−0246 vortex oscillator (Scientific Industries, Bohemia, NY, USA), the reaction was carried out in SHJ−4CD water bath (Changzhou, China) at 70 °C for 30 min, cooled quickly to room temperature, then add 200 µL of hydrochloric acid (0.3 mol/L) and 2 mL of trichloromethane; vortex mixing for 1 min and let stand for 1 min, pipette the upper solution vortex mixing for 1 min and then let stand for 3 min, repeated three times. In total, 2 mL of trichloromethane; vortex mixing for 1 min and let stand for 1 min, remove the upper layer of the solution vortex mixing for 1 min and let stand for 3 min, repeated three times. The upper aqueous phase solution (sample solution after derivatization reaction) was filtered (0.45 µm) to obtain a clarified sample solution (stored at 4 °C). The chromatographic separation was carried out on an Agilent 1260-C18 reversed-phase column (4.6 mm × 250 mm, 5 μm) at 25 °C, with acetonitrile as follows: ammonium acetate buffer solution used as the mobile phase (the concentration was 100 mmol/L, pH 5.5, 22:78, *v*/*v*) at a flow rate of 1 mL/min, and the wavelength of the UV detector was 245 nm; the injection volume was 10 µL. Lactose content and lactose hydrolysis rate were calculated using the following equations.

(1) $$L=\frac{C}{10}\times D$$

(2) $$H(\%)=\frac{m_{1}-m_{0}}{m_{0}}\times 100$$

Formula: *L*—lactose content, g/100 g; *C*—lactose concentration, g/100 g; *D*—dilution factor, 25; *H*—lactose hydrolysis ratio, %; *m*_0_—lactose content in unhydrolyzed samples, g; *m*_1_—lactose content in hydrolyzed samples, g.

#### 3.2.4. Pasteurization of LFM

Samples of LFM (hydrolysis temperature of 38 °C, lactase addition of 0.03%, hydrolysis time of 2.5 h) were pasteurized. LTLT: the temperature was 60 and 65 °C, the time was 20, 25, 30, 35, and 40 min; HTST: temperatures of 70, 75, 80, 85, and 90 °C for 15 s and 30 s.

#### 3.2.5. Analysis of Storage Conditions for LFM Samples

Each sample in “Section 3.2.4” was stored in refrigerated (4 °C), simulated room temperature (25 °C) and high temperature environment (37 °C), and the changes in furosine, HMF, color, and endogenous FI were determined periodically to investigate the effects of different pasteurization conditions on the MR of LFM.

#### 3.2.6. Furosine Content Analysis

The content of furosine in LFM was determined according to NY/T 939-2016 “Identification of restored Milk in Pasteurized Milk and UHT sterilized milk”. Agilent 1260 liquid chromatograph with UV detector (Agilent Technology Co., LTD, Santa Clara, CA, USA) was used to determine furosine content in samples. Unpasteurized LFM was used as a control group. In total, 1 mL of furosine standard (100 μg/mL) was pipetted, diluted with hydrochloric acid solution (3 mol/L), fixed to 50 mL, and mixed well to obtain furosine standard working solution (2 mg/L). In total, 6 mL of hydrochloric acid solution (10.6 mol/L) was added to LFM (2 mL), vortexed and mixed, and then hydrolyzed in a constant temperature DHG−9140A blast drying oven (Shanghai Yiheng Technology Co., LTD, Shanghai, China) at 110 °C (23 h), and then cooled to room temperature after the end of the process, and the hydrolysate was filtered (0.22 μm aqueous phase filter membrane). Then Kjeldahl method was used to determine the protein content in the hydrolysate, copper sulfate (0.4 g) and potassium sulfate (6 g) were added to the hydrolysate (2 mL), mixed, and then sulfuric acid (20 mL) was added, and put into the KDN−19C digesting oven (Shanghai, China) (420 °C) and kept for 1 h. At the end of the process, it was cooled down and added with 50 mL of water, and then it was put on the machine to be measured.

The furosine content of the samples was analyzed using an Agilent 1260-C18 reverse chromatography column (4.6 mm × 250 mm, 5 μm). To the hydrolyzed solution (1 mL), 5 mL of ammonium acetate solution (6 g/L) was added, vortexed, mixed, and filtered (0.22 μm). The mobile phase A was methanol and B was a solution containing 0.1% trifluoroacetic acid with gradient elution (Table 3). The wavelength of the UV detector was 280 nm; the column temperature was 32 °C; and the injection volume was 10 µL. The RSD was 1.38%, which showed good precision. The furosine content was calculated using the following formula.

(3) $$F=\frac{A_{t}\times C_{\mathrm{std}}\times D\times 100}{A_{\mathrm{std}}\times m}\times 100$$

Formula: *F*—lactose content in LFM, mg/100 g protein; *A_t_*—furosine peak area; *C_std_*—concentration of furosine standard solution, 2 mg/L; *D*—dilution factor, 6; *A_std_*—furosine standard solution peak area, 75.9; *m*—protein concentration in hydrolysate, g/L.

#### 3.2.7. HMF Analysis

Refer to NY/T 1332-2007 “Determination of HMF content in milk and dairy products by high-performance liquid chromatography” for determination of HMF content in LFM. Agilent 1260 liquid chromatograph with UV detector (Santa Clara, CA, USA) was used to determine HMF content in samples. Unpasteurized LFM was used as a control group. LFM (10 g) was weighed and added to 5 mL of oxalic acid solution (0.15 mol/L) and heated in a boiling water bath (25 min), at the end of which it was quickly cooled to room temperature in an ice water bath. Then, the solution was made up to 50 mL with methanol (5 mL) (repeated twice), 3 mL of potassium ferricyanide solution (92 g/L) and 3 mL of zinc acetate solution (183 g/L) were added, and the solution was left to stand for 15 min after vigorous shaking, and then filtered through medium-speed filter paper (the first 3 drops of filtrate were discarded), and the filtrate was filtered (0.45 μm). The chromatographic analysis was performed on an Agilent 1260-C18 reversed-phase column (4.6 mm × 250 mm, 5 μm) at 25 °C, with methanol:water (15:85, *v*/*v*) as the mobile phase; the flow rate was 1 mL/min; the wavelength of UV detector was 280 nm; and the injection volume was 10 µL. The RSD was 0.39% with good precision. The content of HMF was calculated according to the following formula.

(4) $$X=\frac{A_{i}}{A_{s}}\times C_{s}\times \frac{V}{m}$$

Formula: *X*—HMF in the sample, mg/kg; *A_i_*—Fpeak area; *A_s_*—peak area of standard solution, 376.3; *C_s_*—standard solution concentration, 3 ug/mL; *V*—sample volume, mL; *m*—sample quality, g.

#### 3.2.8. Color Analysis

Color was determined as previously described [54]. Color measurements were obtained using Konica Minolta CR−400 colorimeter (Konica Minolta Co., LTD, Tokyo, Japan), the color parameters were L* (lightness), the smaller of which indicates that the sample was darker; a* (redness/greenness), a positive value of which indicates that the sample was reddish, while a negative value indicates that the sample was greenish; and b* (yellowness/blueness), a positive value of which indicates that the sample was yellowness, while a negative value indicates that the sample is greenish.

#### 3.2.9. EFS Analysis

EFS was determined as previously described [55], with some modifications. The assay was performed at 25 °C using an F−320 fluorescence spectrophotometer (Tianjin, China). Lactose-free buttermilk (100 μL) was diluted to 20 mL with deionized water, mixed well, and put on the machine for measurement. The excitation wavelength of the fluorescence spectrum was 290 nm (slit 5 nm), and the emission spectrum was collected in the range of 270~460 nm (slit 5 nm), with a scan rate of 1200 nm/min.

### 3.3. Statistical Analysis

Data were processed and analyzed using SPSS 26.0 and plotted using Origin 2021. All experimental data were averaged over three replicates, and results were expressed as mean ± standard deviation.

## 4. Conclusions

By adding free lactase (enzyme activity 5000 NLU/mL), the optimal lactose hydrolysis conditions were determined by single-factor and orthogonal tests as follows: The results showed that the hydrolysis temperature was 38 °C, the amount of lactase added was 0.03%, the hydrolysis time was 2.5 h, and the lactose hydrolysis rate was 97.08% under these conditions. The lactose residue after hydrolysis was only 0.15 g/100 g, which was in line with the standard of LFM in GB 28050-2011. During lactose hydrolysis, the hydrolysis rate was most affected by the hydrolysis temperature and least affected by the hydrolysis time. LFM was heat-treated using pasteurization and stored at 4, 25, and 37 °C, while changes in furosine, HMF, color, and endogenous fluorescence were determined periodically. The contents of furosine and HFM tended to increase after pasteurization of LFM, in addition to changes in color and maximum fluorescence intensity of endogenous fluorescence spectra. As the storage temperature increased, not only did it accelerate the rate of furosine and HMF production in LFM, but also the colors (L*, a*, and b*) were altered and the maximum fluorescence intensity of the endogenous fluorescence spectra was reduced. The results showed that both pasteurization and storage temperature had a great influence on the Maillard reaction of LFM. This study could provide theoretical basis and guidance for the screening of pasteurization conditions and the determination of storage temperature for the industrial production of LFM.

## Figures and Tables

**Table 1:** Results of orthogonal tests for the preparation of LFM by lactase hydrolysis.

Number	A: Hydrolysis Temperature (°C)	B: Lactase Addition (%)	C: Hydrolysis Time (h)	Lactose Hydrolysis Rate (%)
1	1	1	1	59.96
2	2	1	2	80.14
3	3	1	3	79.78
4	1	2	2	79.74
5	2	2	3	91.28
6	3	2	1	91.25
7	1	3	3	92.38
8	2	3	1	92.58
9	3	3	2	92.64
K_1_	219.88	232.08	243.79	
K_2_	262.27	264	252.52	∑759.76
K_3_	277.6	263.67	263.44	
k_1_	73.29	77.36	81.26	
k_2_	87.42	88	84.17	
k_3_	92.53	87.89	87.81	
R	19.24	10.64	6.55	

**Table 2:** Table of orthogonal factor levels.

Level		Considerations	
	A: Hydrolysis Temperature (°C)	B: Lactase Addition (%)	C: Hydrolysis Time (h)
1	36	0.02	1.5
2	37	0.025	2
3	38	0.03	2.5

**Table 3:** Furosine gradient elution table.

Number	Time (min)	Mobile Phase A (%)	Mobile Phase B (%)	Flow Rates (mL/min)
1	0	100	0	1
2	16	86.8	13.2	1
3	16.5	0	100	1
4	25	100	0	1
5	30	100	0	1

## Data Availability

Not applicable.

## Appendix A

**Table A1:** The color change in LFM after pasteurization.

Pasteurization Conditions (°C—min)	L*	a*	b*
Control	107.39 ± 0.85 ^a^	0.21 ± 0.03 ^a^	8.62 ± 0.83 ^a^
60–20	93.86 ± 0.51 ^hijkl^	−0.44 ± 0.07 ^gh^	3.44 ± 0.37 ^fgh^
60–25	88.11 ± 0.46 ^n^	−0.10 ± 0.07 ^bc^	1.88 ± 0.10 ^i^
60–30	90.58 ± 0.16 ^mn^	−0.47 ± 0.01 ^gh^	3.56 ± 0.09 ^fgh^
60–35	95.48 ± 0.34 ^ghi^	−0.26 ± 0.03 ^de^	3.84 ± 0.10 ^efg^
60–40	101.73 ± 0.61 ^bck^	−0.69 ± 0.06 ^k^	5.11 ± 0.10 ^bc^
65–20	94.38 ± 0.74 ^ghij^	−0.52 ± 0.03 ^hi^	3.55 ± 0.20 ^fgh^
65–25	95.41 ± 0.91 ^ghi^	−0.03 ± 0.05 ^b^	3.77 ± 0.23 ^efg^
65–30	92.72 ± 0.47 ^jklm^	−0.25 ± 0.02 ^de^	2.57 ± 0.11 ^hi^
65–35	95.95 ± 0.19 ^fghi^	−0.35 ± 0.01 ^efg^	3.57 ± 0.07 ^fg^
65–40	94.76 ± 1.68 ^ghijk^	−0.10 ± 0.07 ^bcj^	4.30 ± 0.53 ^cdef^
**Pasteurization** **conditions (°C—s)**	**L***	**a***	**b***
70–15	99.61 ± 0.22 ^bcde^	−0.80 ± 0.01 ^kl^	5.48 ± 0.05 ^b^
70–30	91.41 ± 0.35 ^lm^	−0.38 ± 0.02 ^efg^	3.76 ± 0.09 ^efg^
75–15	98.82 ± 2.90 ^de^	−0.43 ± 0.09 ^fgh^	5.29 ± 0.26 ^b^
75–30	98.67 ± 0.45 ^def^	−0.31 ± 0.04 ^def^	4.84 ± 0.10 ^bcd^
80–15	92.36 ± 0.54 ^klm^	−0.65 ± 0.02 ^ij^	4.71 ± 0.09 ^bcde^
80–30	92.53 ± 0.29 ^djklm^	−0.29 ± 0.02 ^de^	2.86 ± 0.06 ^ghi^
85–15	96.77 ± 0.41 ^efg^	−0.68 ± 0.09 ^jk^	5.47 ± 0.62 ^b^
85–30	99.20 ± 0.17 ^ce^	−0.30 ± 0.02 ^de^	4.05 ± 0.08 ^def^
90–15	102.35 ± 0.70 ^b^	−0.83 ± 0.03 ^l^	5.65 ± 0.24 ^b^
90–30	95.20 ± 0.76 ^ghij^	−0.18 ± 0.04 ^cd^	4.01 ± 0.54 ^def^

L*, lightness; a*, greenness/redness; b*, blueness/yellowness; data represent mean values ± SDs. Different lowercase letters in a column of each stage indicate significant differences (*p* < 0.05).

**Table A2:** The color change in LFM after pasteurization during storage at 4 °C.

Pasteurization Conditions (°C—min)	Storage Time (d)	L*	a*	b*
60–20	0	93.86 ± 0.51 ^c^	−0.44 ± 0.07 ^a^	3.44 ± 0.37 ^b^
	3	93.07 ± 0.31 ^c^	−0.72 ± 0.02 ^c^	3.04 ± 0.04 ^c^
	6	98.65 ± 1.51 ^b^	−0.68 ± 0.08 ^c^	4.35 ± 0.25 ^a^
	9	100.96 ± 1.01 ^a^	−0.39 ± 0.02 ^a^	4.69 ± 0.15 ^a^
	12	90.63 ± 0.14 ^d^	−0.69 ± 0.03 ^c^	3.65 ± 0.11 ^b^
	15	91.40 ± 0.27 ^d^	−0.53 ± 0.02 ^b^	2.41 ± 0.09 ^d^
60–25	0	88.11 ± 0.46 ^f^	−0.10 ± 0.07 ^a^	1.88 ± 0.10 d
	3	93.41 ± 0.35 ^c^	−0.79 ± 0.04 ^e^	3.62 ± 0.12 ^bc^
	6	95.17 ± 0.32 ^b^	−0.45 ± 0.02 ^c^	3.31 ± 0.19 ^b^
	9	92.11 ± 0.28 ^d^	−0.26 ± 0.02 ^b^	1.55 ± 0.06 ^d^
	12	90.49 ± 0.11 ^e^	−0.60 ± 0.03 ^d^	2.82 ± 0.64 ^c^
	15	99.14 ± 0.24 ^a^	−0.59 ± 0.02 ^d^	4.35 ± 0.08 ^a^
60–30	0	90.58 ± 0.16 ^d^	−0.47 ± 0.01 a	3.56 ± 0.09 ^bc^
	3	95.30 ± 0.20 ^b^	−0.91 ± 0.07 ^d^	3.91 ± 0.05 ^a^
	6	98.52 ± 0.11 ^a^	−0.57 ± 0.02 ^b^	2.88 ± 0.07 ^d^
	9	95.27 ± 0.34 ^b^	−0.41 ± 0.03 ^a^	3.50 ± 0.25 ^c^
	12	97.41 ± 0.30 ^a^	−0.70 ± 0.01 ^c^	3.45 ± 0.12 ^c^
	15	92.93 ± 1.49 ^c^	−0.73 ± 0.06 ^c^	3.84 ± 0.27 ^ab^
60–35	0	95.48 ± 0.34 ^c^	−0.26 ± 0.03 ^a^	3.84 ± 0.10 ^b^
	3	96.38 ± 0.33 ^b^	−1.15 ± 0.03 ^d^	3.95 ± 0.10 ^b^
	6	98.68 ± 0.21 ^a^	−0.76 ± 0.01 ^c^	3.23 ± 0.07 ^c^
	9	96.63 ± 0.41 ^b^	−0.29 ± 0.03 ^a^	4.63 ± 0.07 ^a^
	12	93.47 ± 0.75 ^d^	−0.46 ± 0.05 ^b^	2.86 ± 0.12 ^d^
	15	94.68 ± 0.51 ^c^	−0.73 ± 0.02 ^c^	2.53 ± 0.11 ^e^
60–40	0	101.73 ± 0.61 ^a^	−0.69 ± 0.06 ^bc^	5.11 ± 0.10 ^a^
	3	102.63 ± 0.32 ^a^	−1.04 ± 0.05 ^e^	4.76 ± 0.38 ^ab^
	6	93.51 ± 0.88 ^c^	−0.88 ± 0.01 ^d^	4.52 ± 0.18 ^b^
	9	94.97 ± 0.46 ^b^	−0.52 ± 0.03 ^a^	4.08 ± 0.17 ^c^
	12	93.36 ± 0.48 ^c^	−0.62 ± 0.02 ^b^	3.12 ± 0.19 ^d^
	15	93.16 ± 0.57 ^c^	−0.71 ± 0.02 ^c^	3.25 ± 0.10 ^d^
65–25	0	95.41 ± 0.91 ^c^	−0.03 ± 0.05 ^a^	3.77 ± 0.23 ^bc^
	3	98.96 ± 0.29 ^b^	−0.92 ± 0.02 ^d^	4.23 ± 0.24 ^b^
	6	102.08 ± 1.09 ^a^	−1.00 ± 0.09 ^d^	5.57 ± 0.59 ^a^
	9	92.82 ± 1.06 ^d^	−0.68 ± 0.04 ^c^	3.46 ± 0.34 ^c^
	12	98.57 ± 1.47 ^b^	−0.48 ± 0.04 ^b^	3.64 ± 0.24 ^bc^
	15	96.12 ± 0.77 ^c^	−0.66 ± 0.04 ^c^	3.37 ± 0.13 ^c^
65–30	0	92.72 ± 0.47 ^c^	−0.25 ± 0.02 ^a^	2.57 ± 0.11 ^d^
	3	87.18 ± 1.30 ^d^	−0.51 ± 0.04 ^c^	1.47 ± 0.29 ^e^
	6	92.46 ± 0.71 ^c^	−0.64 ± 0.04 ^d^	3.66 ± 0.24 ^a^
	9	97.02 ± 0.42 ^b^	−0.68 ± 0.04 ^d^	3.59 ± 0.13 ^ab^
	12	96.46 ± 0.71 ^b^	−0.80 ± 0.02 ^e^	3.28 ± 0.06 ^bc^
	15	98.61 ± 0.22 ^a^	−0.41 ± 0.03 ^b^	2.99 ± 0.13 ^c^
65–35	0	95.95 ± 0.19 ^e^	−0.35 ± 0.01 ^b^	3.57 ± 0.07 ^e^
	3	99.91 ± 0.87 ^c^	−1.17 ± 0.02 ^e^	5.70 ± 0.21 ^a^
	6	102.86 ± 0.35 ^b^	−1.13 ± 0.02 ^de^	4.87 ± 0.10 ^c^
	9	103.82 ± 0.20 ^a^	−1.09 ± 0.04 ^d^	5.35 ± 0.18 ^b^
	12	98.67 ± 0.12 ^d^	−0.68 ± 0.02 ^c^	3.83 ± 0.06 ^d^
	15	94.29 ± 0.58 ^f^	−0.24 ± 0.04 ^a^	2.61 ± 0.16 ^f^
65–40	0	94.76 ± 1.68 ^c^	−0.10 ± 0.07 ^a^	4.30 ± 0.53 ^ab^
	3	95.66 ± 0.90 ^bc^	−0.44 ± 0.03 ^b^	3.53 ± 0.25 ^cd^
	6	93.72 ± 1.05 ^c^	−0.63 ± 0.03 ^c^	3.27 ± 0.24 ^d^
	9	98.66 ± 2.04 ^a^	−0.66 ± 0.05 ^cd^	4.40 ± 0.20 ^a^
	12	98.26 ± 0.27 ^a^	−0.65 ± 0.04 ^cd^	3.15 ± 0.15 ^d^
	15	97.18 ± 0.30 ^ab^	−0.72 ± 0.02 ^d^	3.85 ± 0.07 ^bc^
**Pasteurization** **conditions** **(°C—s)**	**Storage** **time (d)**	**L***	**a***	**b***
70–15	0	99.61 ± 0.22 ^a^	−0.80 ± 0.01 ^b^	5.48 ± 0.05 ^a^
	3	95.55 ± 0.25 ^b^	−0.84 ± 0.03 ^b^	4.59 ± 0.09 ^b^
	6	92.76 ± 2.20 ^cd^	−0.84 ± 0.05 ^b^	3.48 ± 0.47 ^cd^
	9	94.46 ± 0.88 ^bc^	−0.95 ± 0.06 ^c^	5.12 ± 0.56 ^ab^
	12	91.37 ± 1.13 ^d^	−0.51 ± 0.07 ^a^	2.84 ± 0.53 ^d^
	15	96.34 ± 0.06 ^b^	−0.43 ± 0.01 ^a^	3.88 ± 0.03 ^c^
70–30	0	91.41 ± 0.35 ^e^	−0.38 ± 0.02 ^b^	3.76 ± 0.09 ^b^
	3	95.81 ± 0.62 ^c^	−1.05 ± 0.03 ^f^	3.75 ± 0.13 ^b^
	6	93.71 ± 0.75 ^d^	−0.65 ± 0.04 ^c^	2.12 ± 0.29 ^c^
	9	96.64 ± 0.03 ^b^	−0.70 ± 0.02 ^d^	3.64 ± 0.07 ^b^
	12	99.12 ± 0.25 ^a^	−0.96 ± 0.01 ^e^	4.56 ± 0.07 ^a^
	15	86.82 ± 0.08 ^f^	−0.27 ± 0.02 ^a^	1.49 ± 0.07 ^d^
75–15	0	98.82 ± 2.90 ^ab^	−0.43 ± 0.09 ^b^	5.29 ± 0.26 ^a^
	3	91.12 ± 0.06 ^cd^	−0.77 ± 0.01 ^c^	2.97 ± 0.02 ^d^
	6	100.43 ± 1.78 ^a^	−0.75 ± 0.08 ^c^	4.59 ± 0.19 ^b^
	9	88.64 ± 0.50 ^d^	−0.53 ± 0.03 ^b^	2.90 ± 0.30 ^d^
	12	92.64 ± 0.39 ^c^	−0.14 ± 0.05 ^a^	2.34 ± 0.55 ^e^
	15	96.17 ± 2.09 ^b^	−0.44 ± 0.08 ^b^	3.65 ± 0.17 ^c^
75–30	0	98.67 ± 0.45 ^c^	−0.31 ± 0.04 ^a^	4.84 ± 0.10 ^b^
	3	102.87 ± 0.06 ^a^	−1.13 ± 0.04 ^e^	5.35 ± 0.12 ^a^
	6	96.53 ± 0.78 ^e^	−0.78 ± 0.03 ^c^	3.37 ± 0.21 ^e^
	9	97.59 ± 0.18 ^d^	−0.81 ± 0.03 ^c^	3.72 ± 0.14 ^d^
	12	101.67 ± 0.46 ^b^	−1.00 ± 0.02 ^d^	4.48 ± 0.19 ^c^
	15	91.80 ± 0.39 ^f^	−0.42 ± 0.03 ^b^	3.79 ± 0.12 ^d^
80–15	0	92.36 ± 0.54 ^c^	−0.65 ± 0.02 ^b^	4.71 ± 0.09 ^ab^
	3	94.90 ± 0.26 ^b^	−0.93 ± 0.02 ^d^	4.42 ± 0.06 ^b^
	6	94.93 ± 0.19 ^b^	−0.74 ± 0.04 ^c^	4.61 ± 0.02 ^ab^
	9	96.99 ± 0.58 ^a^	−0.66 ± 0.06 ^b^	4.88 ± 0.43 ^a^
	12	94.40 ± 0.55 ^b^	−0.95 ± 0.01 ^d^	3.59 ± 0.13 ^c^
	15	89.35 ± 0.47 ^d^	−0.35 ± 0.04 ^a^	1.68 ± 0.19 ^d^
80–30	0	92.53 ± 0.29 ^c^	−0.29 ± 0.02 ^a^	2.86 ± 0.06 ^bc^
	3	91.37 ± 0.27 ^de^	−0.54 ± 0.01 ^bc^	2.18 ± 0.10 ^d^
	6	97.05 ± 0.37 ^a^	−0.79 ± 0.06 ^e^	3.49 ± 0.21 ^a^
	9	92.24 ± 0.19 ^cd^	−0.69 ± 0.03 ^d^	3.26 ± 0.14 ^ab^
	12	90.48 ± 1.41 ^e^	−0.46 ± 0.08 ^b^	2.11 ± 0.52 ^d^
	15	95.77 ± 0.05 ^b^	−0.57 ± 0.03 ^c^	2.56 ± 0.04 ^cd^
85–15	0	96.77 ± 0.41 ^b^	−0.68 ± 0.09 ^c^	5.47 ± 0.62 ^a^
	3	100.76 ± 0.51 ^a^	−0.87 ± 0.01 ^d^	5.08 ± 0.36 ^a^
	6	92.63 ± 1.22 ^d^	−0.91 ± 0.04 ^d^	3.77 ± 0.14 ^b^
	9	90.81 ± 0.55 ^e^	−0.32 ± 0.02 ^a^	3.35 ± 0.15 ^b^
	12	94.04 ± 0.56 ^c^	−0.29 ± 0.01 ^a^	2.67 ± 0.13 ^c^
	15	94.40 ± 0.06 ^c^	−0.54 ± 0.01 ^b^	2.67 ± 0.03 ^c^
85–30	0	99.20 ± 0.17 ^bc^	−0.30 ± 0.02 ^a^	4.05 ± 0.08 ^b^
	3	98.28 ± 0.41 ^c^	−1.05 ± 0.04 ^f^	4.16 ± 0.15 ^b^
	6	89.09 ± 0.68 ^d^	−0.56 ± 0.02 ^c^	2.78 ± 0.14 ^c^
	9	99.70 ± 0.18 ^b^	−0.70 ± 0.03 ^d^	3.92 ± 0.29 ^b^
	12	102.41 ± 0.20 ^a^	−0.91 ± 0.02 ^e^	4.70 ± 0.05 ^a^
	15	101.97 ± 1.65 ^a^	−0.49 ± 0.05 ^b^	3.99 ± 0.14 ^b^
90–15	0	102.35 ± 0.70 ^b^	−0.83 ± 0.03 ^d^	5.65 ± 0.24 ^a^
	3	99.34 ± 0.60 ^c^	−0.68 ± 0.02 ^c^	4.99 ± 0.04 ^b^
	6	95.28 ± 0.21 ^d^	−0.56 ± 0.04 ^b^	4.47 ± 0.09 ^c^
	9	93.48 ± 0.38 ^e^	−0.48 ± 0.13 ^b^	2.92 ± 0.04 ^d^
	12	103.60 ± 0.04 ^a^	−0.89 ± 0.05 ^d^	4.30 ± 0.01 ^c^
	15	88.41 ± 0.84 ^f^	−0.04 ± 0.04 ^a^	1.86 ± 0.42 ^e^
90–30	0	95.20 ± 0.76 ^c^	−0.18 ± 0.04 ^a^	4.01 ± 0.54 ^a^
	3	98.03 ± 0.17 ^b^	−0.73 ± 0.02 ^d^	3.30 ± 0.02 ^b^
	6	95.08 ± 0.29 ^c^	−0.77 ± 0.08 ^d^	3.70 ± 0.44 ^ab^
	9	90.00 ± 0.62 ^e^	−0.64 ± 0.03 ^c^	2.43 ± 0.15 ^c^
	12	99.24 ± 0.20 ^a^	−0.79 ± 0.03 ^d^	3.84 ± 0.20 ^ab^
	15	92.42 ± 0.12 ^d^	−0.55 ± 0.02 ^b^	1.86 ± 0.07 ^d^

L*, lightness; a*, greenness/redness; b*, blueness/yellowness; data represent mean values ± SDs. Different lowercase letters in a column of each stage indicate significant differences (*p* < 0.05).

**Table A3:** The color change in LFM after pasteurization during storage at 25 °C.

Pasteurization Conditions (°C—min)	Storage Time (d)	L*	a*	b*
60–20	0	93.86 ± 0.51 ^a^	−0.44 ± 0.07 ^a^	3.44 ± 0.37 ^b^
	2	82.47 ± 0.89 ^d^	−0.73 ± 0.02 ^b^	2.24 ± 0.16 ^cd^
	4	88.48 ± 1.00 ^b^	−1.11 ± 0.10 ^c^	2.51 ± 0.53 ^c^
	6	79.38 ± 0.35 ^e^	−0.47 ± 0.05 ^a^	1.83 ± 0.14 ^d^
	8	80.30 ± 1.64 ^de^	−1.31 ± 0.06 ^d^	4.41 ± 0.32 ^a^
	10	84.95 ± 2.29 ^c^	−1.14 ± 0.05 ^c^	3.59 ± 0.40 ^b^
60–25	0	88.11 ± 0.46 ^bc^	−0.10 ± 0.07 ^a^	1.88 ± 0.10 ^d^
	2	95.28 ± 2.27 ^a^	−0.44 ± 0.09 ^b^	3.87 ± 0.60 ^b^
	4	89.11 ± 2.31 ^b^	−0.17 ± 0.07 ^a^	2.99 ± 0.49 ^c^
	6	87.83 ± 0.37 ^bc^	−0.63 ± 0.02 ^c^	4.84 ± 0.14 ^a^
	8	85.96 ± 0.22 ^cd^	−0.62 ± 0.04 ^c^	4.02 ± 0.13 ^b^
	10	84.18 ± 1.30 ^d^	−0.80 ± 0.03 ^d^	3.50 ± 0.27 ^bc^
60–30	0	90.58 ± 0.16 ^a^	−0.47 ± 0.01 ^a^	3.56 ± 0.09 ^ab^
	2	82.51 ± 0.33 ^c^	−0.50 ± 0.04 ^a^	1.67 ± 0.02 ^d^
	4	84.43 ± 0.79 ^b^	−0.90 ± 0.03 ^b^	2.40 ± 0.10 ^c^
	6	80.90 ± 0.48 ^d^	−1.13 ± 0.03 ^c^	3.74 ± 0.13 ^a^
	8	82.64 ± 0.67 ^c^	−0.96 ± 0.05 ^b^	2.59 ± 0.14 ^c^
	10	74.82 ± 1.22 ^e^	−1.13 ± 0.04 ^c^	3.25 ± 0.51 ^b^
60–35	0	95.48 ± 0.34 ^a^	−0.26 ± 0.03 ^a^	3.84 ± 0.10 ^bc^
	2	94.62 ± 0.28 ^ab^	−0.27 ± 0.07 ^a^	4.04 ± 0.28 ^b^
	4	92.24 ± 0.19 ^c^	−0.30 ± 0.01 ^a^	3.99 ± 0.04 ^b^
	6	89.18 ± 0.39 ^d^	−0.41 ± 0.03 ^b^	3.55 ± 0.17 ^c^
	8	94.31 ± 0.36 ^b^	−0.43 ± 0.03 ^b^	3.78 ± 0.08 ^bc^
	10	95.10 ± 0.84 ^ab^	−1.01 ± 0.07 ^c^	6.13 ± 0.27 ^a^
60–40	0	101.73 ± 0.61 ^a^	−0.69 ± 0.06 c	5.11 ± 0.10 a
	2	78.01 ± 0.32 ^cd^	−0.16 ± 0.02 ^a^	2.01 ± 0.06 ^d^
	4	80.66 ± 1.45 ^bc^	−0.70 ± 0.03 ^c^	2.19 ± 0.26 ^cd^
	6	83.43 ± 1.65 ^b^	−0.69 ± 0.06 ^c^	2.58 ± 0.36 ^c^
	8	77.02 ± 2.86 ^d^	−0.52 ± 0.12 ^b^	1.44 ± 0.52 ^e^
	10	83.32 ± 1.34 ^b^	−1.09 ± 0.04 ^d^	3.18 ± 0.26 ^b^
65–20	0	94.38 ± 0.74 ^b^	−0.52 ± 0.03 ^a^	3.55 ± 0.20 ^c^
	2	99.11 ± 1.06 ^a^	−0.74 ± 0.03 ^c^	5.12 ± 0.28 ^a^
	4	87.25 ± 0.24 ^c^	−0.53 ± 0.06 ^a^	3.90 ± 0.12 ^b^
	6	93.89 ± 1.35 ^b^	−0.64 ± 0.03 ^b^	5.21 ± 0.36 ^a^
	8	82.70 ± 0.16 ^d^	−0.52 ± 0.04 ^a^	3.16 ± 0.13 ^c^
	10	83.32 ± 1.15 ^d^	−0.51 ± 0.05 ^a^	3.13 ± 0.22 ^c^
65–25	0	95.41 ± 0.91 ^a^	−0.03 ± 0.05 ^a^	3.77 ± 0.23 ^c^
	2	92.68 ± 0.19 ^b^	−0.40 ± 0.01 ^c^	4.06 ± 0.06 ^b^
	4	90.43 ± 0.09 ^c^	−0.44 ± 0.01 ^cd^	3.34 ± 0.02 ^d^
	6	91.03 ± 0.29 ^c^	−0.46 ± 0.02 ^d^	4.05 ± 0.02 ^b^
	8	80.57 ± 0.35 ^e^	−0.24 ± 0.02 ^b^	1.27 ± 0.07 ^e^
	10	89.49 ± 0.23 ^d^	−0.58 ± 0.01 ^e^	4.95 ± 0.15 ^a^
65–30	0	92.72 ± 0.47 ^a^	−0.25 ± 0.02 ^b^	2.57 ± 0.11 ^d^
	2	85.40 ± 0.64 ^c^	−0.08 ± 0.05 ^a^	3.33 ± 0.26 ^c^
	4	92.81 ± 0.87 ^a^	−0.80 ± 0.02 ^d^	5.68 ± 0.20 ^a^
	6	85.80 ± 0.4 ^c^	−0.47 ± 0.02 ^c^	3.22 ± 0.05 ^c^
	8	87.78 ± 0.36 ^b^	−0.90 ± 0.03 ^e^	4.39 ± 0.07 ^b^
	10	72.09 ± 0.63 ^d^	−0.82 ± 0.02 ^d^	2.77 ± 0.17 ^d^
65–35	0	95.95 ± 0.19 ^c^	−0.35 ± 0.01 ^a^	3.57 ± 0.07 ^c^
	2	99.12 ± 0.36 ^a^	−0.61 ± 0.02 ^b^	4.34 ± 0.16 ^b^
	4	98.04 ± 0.18 ^b^	−0.56 ± 0.01 ^b^	3.43 ± 0.04 ^c^
	6	95.88 ± 0.50 ^c^	−0.88 ± 0.01 ^c^	4.77 ± 0.28 ^b^
	8	82.17 ± 0.07 ^e^	−0.54 ± 0.04 ^b^	2.87 ± 0.23 ^d^
	10	84.78 ± 1.24 ^d^	−1.60 ± 0.09 ^d^	5.33 ± 0.54 ^a^
65–40	0	94.76 ± 1.68 ^a^	−0.10 ± 0.07 ^a^	4.30 ± 0.53 ^b^
	2	85.84 ± 0.32 ^b^	−0.55 ± 0.03 ^b^	3.85 ± 0.04 ^bc^
	4	84.19 ± 0.26 ^b^	−0.71 ± 0.01 ^c^	2.98 ± 0.06 ^d^
	6	80.10 ± 3.13 ^c^	−0.73 ± 0.04 ^c^	3.42 ± 0.30 ^cd^
	8	83.93 ± 0.93 ^b^	−0.91 ± 0.11 ^d^	4.92 ± 0.27 ^a^
	10	85.54 ± 1.27 ^b^	−1.06 ± 0.06 ^e^	4.25 ± 0.25 ^b^
**Pasteurization** **conditions** **(°C—s)**	**Storage** **time (d)**	**L***	**a***	**b***
70–15	0	99.61 ± 0.22 ^a^	−0.80 ± 0.01 ^a^	5.48 ± 0.05 ^a^
	2	91.17 ± 1.13 ^b^	−1.05 ± 0.05 ^b^	4.67 ± 0.29 ^bc^
	4	87.78 ± 0.97 ^c^	−0.67 ± 0.06 ^a^	4.20 ± 0.27 ^cd^
	6	82.26 ± 2.94 ^d^	−1.21 ± 0.11 ^c^	4.87 ± 0.57 ^b^
	8	84.76 ± 0.74 ^d^	−0.98 ± 0.06 ^b^	4.43 ± 0.07 ^bcd^
	10	76.65 ± 1.99 ^e^	−0.80 ± 0.13 ^a^	4.00 ± 0.20 ^d^
70–30	0	91.41 ± 0.35 ^b^	−0.38 ± 0.02 ^b^	3.76 ± 0.09 ^b^
	2	97.15 ± 0.24 ^a^	−0.30 ± 0.01 ^a^	5.28 ± 0.19 ^a^
	4	83.82 ± 0.53 ^e^	−0.31 ± 0.03 ^a^	2.83 ± 0.15 ^b^
	6	86.65 ± 0.17 ^d^	−0.79 ± 0.03 ^c^	3.42 ± 0.15 ^b^
	8	89.77 ± 0.77 ^c^	−1.24 ± 0.03 ^e^	5.53 ± 0.19 ^a^
	10	81.55 ± 1.28 ^f^	−0.92 ± 0.06 ^d^	4.78 ± 1.36 ^a^
75–15	0	98.82 ± 2.90 ^a^	−0.43 ± 0.09 ^b^	5.29 ± 0.26 ^b^
	2	94.06 ± 1.45 ^b^	−0.54 ± 0.04 ^c^	5.78 ± 0.26 ^a^
	4	94.08 ± 0.27 ^b^	−0.48 ± 0.03 ^bc^	3.88 ± 0.06 ^c^
	6	89.83 ± 0.64 ^c^	−0.05 ± 0.02 ^a^	3.52 ± 0.27 ^cd^
	8	91.60 ± 1.23 ^bc^	−0.54 ± 0.06 ^c^	3.23 ± 0.31 ^d^
	10	78.44 ± 0.25 ^d^	−0.57 ± 0.01 ^c^	1.53 ± 0.07 ^e^
75–30	0	98.67 ± 0.45 ^a^	−0.31 ± 0.04 ^a^	4.84 ± 0.10 ^a^
	2	96.31 ± 0.07 ^b^	−0.43 ± 0.02 ^c^	3.70 ± 0.01 ^b^
	4	94.52 ± 0.15 ^c^	−0.62 ± 0.01 ^d^	4.93 ± 0.08 ^a^
	6	92.10 ± 0.54 ^d^	−0.35 ± 0.03 ^b^	2.62 ± 0.16 ^d^
	8	89.39 ± 0.06 ^e^	−0.72 ± 0.03 ^e^	3.75 ± 0.04 ^b^
	10	88.40 ± 0.28 ^f^	−0.83 ± 0.02 ^f^	3.32 ± 0.12 ^c^
80–15	0	92.36 ± 0.54 ^a^	−0.65 ± 0.02 ^c^	4.71 ± 0.09 ^a^
	2	89.12 ± 0.61 ^b^	−0.43 ± 0.02 ^a^	3.70 ± 0.01 ^b^
	4	87.00 ± 2.54 ^b^	−0.81 ± 0.07 ^d^	3.43 ± 0.50 ^b^
	6	79.62 ± 0.87 ^c^	−0.48 ± 0.02 ^ab^	2.72 ± 0.27 ^c^
	8	80.47 ± 0.62 ^c^	−0.54 ± 0.05 ^b^	2.36 ± 0.13 ^c^
	10	82.47 ± 2.46 ^c^	−0.81 ± 0.08 ^d^	3.67 ± 0.58 ^b^
80–30	0	92.53 ± 0.29 ^a^	−0.29 ± 0.02 ^b^	2.86 ± 0.06 ^b^
	2	79.19 ± 0.98 ^cd^	−0.48 ± 0.02 ^d^	2.31 ± 0.22 ^bc^
	4	82.33 ± 0.28 ^b^	−0.38 ± 0.01 ^c^	2.65 ± 0.06 ^bc^
	6	75.25 ± 1.72 ^e^	−0.15 ± 0.03 ^a^	1.43 ± 0.34 ^d^
	8	78.46 ± 0.23 ^d^	−0.73 ± 0.03 ^f^	2.21 ± 0.45 ^c^
	10	80.31 ± 0.90 ^c^	−0.69 ± 0.02 ^e^	3.70 ± 0.46 ^a^
85–15	0	96.77 ± 0.41 ^a^	−0.68 ± 0.09 ^c^	5.47 ± 0.62 ^a^
	2	85.05 ± 0.27 ^c^	−0.52 ± 0.02 ^b^	3.35 ± 0.06 ^c^
	4	95.92 ± 1.53 ^a^	−0.92 ± 0.02 ^e^	4.91 ± 0.41 ^ab^
	6	83.07 ± 0.27 ^d^	−0.38 ± 0.02 ^a^	2.91 ± 0.10 ^c^
	8	84.81 ± 0.26 ^c^	−0.82 ± 0.02 ^d^	4.77 ± 0.10 ^b^
	10	86.93 ± 0.45 ^b^	−0.91 ± 0.02 ^e^	5.07 ± 0.09 ^ab^
85–30	0	99.20 ± 0.17 ^a^	−0.30 ± 0.02 ^ab^	4.05 ± 0.08 ^b^
	2	90.75 ± 0.36 ^b^	−0.48 ± 0.01 ^c^	3.92 ± 0.15 ^bc^
	4	84.98 ± 0.71 ^d^	−0.38 ± 0.04 ^b^	3.58 ± 0.17 ^c^
	6	73.54 ± 0.73 ^e^	−0.23 ± 0.02 ^a^	1.96 ± 0.13 ^d^
	8	88.03 ± 0.56 ^c^	−0.96 ± 0.06 ^e^	5.17 ± 0.31 ^a^
	10	87.34 ± 2.20 ^c^	−0.79 ± 0.11 ^d^	4.34 ± 0.44 ^b^
90–15	0	102.35 ± 0.70 ^a^	−0.83 ± 0.03 ^c^	5.65 ± 0.24 ^a^
	2	87.90 ± 0.39 ^b^	−0.65 ± 0.03 ^b^	4.61 ± 0.06 ^b^
	4	84.24 ± 1.13 ^c^	−0.55 ± 0.02 ^a^	2.92 ± 0.30 ^c^
	6	82.61 ± 1.13 ^d^	−0.86 ± 0.07 ^c^	4.24 ± 0.47 ^b^
	8	81.26 ± 0.12 ^de^	−1.26 ± 0.07 ^d^	4.64 ± 0.03 ^b^
	10	80.59 ± 0.71 ^e^	−0.88 ± 0.02 ^c^	4.67 ± 0.07 ^b^
90–30	0	95.20 ± 0.76 ^a^	−0.18 ± 0.04 ^a^	4.01 ± 0.54 ^b^
	2	87.28 ± 0.71 ^b^	−0.66 ± 0.02 ^d^	4.17 ± 0.20 ^b^
	4	80.00 ± 0.84 ^d^	−0.55 ± 0.01 ^c^	3.42 ± 0.16 ^c^
	6	82.89 ± 2.05 ^c^	−0.64 ± 0.06 ^d^	4.92 ± 0.08 ^a^
	8	78.61 ± 1.88 ^d^	−1.04 ± 0.02 ^e^	3.79 ± 0.37 ^bc^
	10	79.28 ± 0.95 ^d^	−0.44 ± 0.02 ^b^	2.52 ± 0.16 ^d^

L*, lightness; a*, greenness/redness; b*, blueness/yellowness; data represent mean values ± SDs. Different lowercase letters in a column of each stage indicate significant differences (*p* < 0.05).

**Table A4:** The color change in LFM after pasteurization during storage at 37 °C.

Pasteurization Conditions (°C—min)	Storage Time (d)	L*	a*	b*
60–20	0	93.86 ± 0.51 ^a^	−0.44 ± 0.07 ^b^	3.44 ± 0.37 ^c^
	1	68.81 ± 1.07 ^bc^	−1.91 ± 0.02 ^c^	1.65 ± 0.08 ^e^
	2	67.03 ± 1.46 ^cd^	0.36 ± 0.50 ^a^	4.87 ± 0.11 ^a^
	3	68.23 ± 1.05 ^bc^	−1.88 ± 0.10 ^c^	2.38 ± 0.25 ^d^
	4	69.87 ± 1.77 ^b^	−2.35 ± 0.35 ^cd^	4.17 ± 0.32 ^b^
	5	65.62 ± 1.39 ^d^	−2.46 ± 0.24 ^d^	5.20 ± 0.46 ^a^
60–25	0	88.11 ± 0.46 ^a^	−0.10 ± 0.07 ^a^	1.88 ± 0.10 ^d^
	1	72.26 ± 0.49 ^c^	−2.35 ± 0.17 ^c^	2.92 ± 0.17 ^c^
	2	63.75 ± 1.02 ^e^	−0.09 ± 0.09 ^a^	5.02 ± 0.23 ^a^
	3	73.29 ± 1.59 ^bc^	−2.65 ± 0.06 ^d^	4.24 ± 0.37 ^b^
	4	74.12 ± 0.41 ^b^	−2.55 ± 0.05 ^d^	3.27 ± 0.12 ^c^
	5	66.04 ± 0.66 ^d^	−1.65 ± 0.06 ^b^	1.20 ± 0.24 ^e^
60–30	0	90.58 ± 0.16 ^a^	−0.47 ± 0.01 ^a^	3.56 ± 0.09 ^b^
	1	64.01 ± 0.34 ^d^	−1.73 ± 0.23 ^b^	0.54 ± 0.09 ^e^
	2	67.38 ± 0.71 ^c^	−0.32 ± 0.10 ^a^	4.93 ± 0.14 ^a^
	3	70.01 ± 1.48 ^b^	−2.18 ± 0.11 ^c^	2.08 ± 0.30 ^c^
	4	70.54 ± 2.04 ^b^	−2.01 ± 0.17 ^c^	1.67 ± 0.08 ^d^
	5	61.30 ± 0.90 ^e^	−2.10 ± 0.04 ^c^	2.34 ± 0.24 ^c^
60–35	0	95.48 ± 0.34 ^a^	−0.26 ± 0.03 ^b^	3.84 ± 0.10 ^b^
	1	72.30 ± 0.50 ^b^	−2.20 ± 0.13 ^d^	2.46 ± 0.08 ^c^
	2	65.43 ± 0.45 ^d^	0.15 ± 0.15 ^a^	4.37 ± 0.21 ^a^
	3	72.66 ± 0.59 ^b^	−2.28 ± 0.10 ^d^	2.07 ± 0.09 ^d^
	4	67.40 ± 1.08 ^c^	−2.02 ± 0.27 ^cd^	2.25 ± 0.34 ^cd^
	5	68.18 ± 0.97 ^c^	−1.81 ± 0.19 ^c^	2.54 ± 0.04 ^c^
60–40	0	101.73 ± 0.61 ^a^	−0.69 ± 0.06 ^b^	5.11 ± 0.10 ^b^
	1	67.70 ± 0.61 ^bc^	−1.64 ± 0.08 ^c^	1.55 ± 0.08 ^c^
	2	66.00 ± 1.41 ^c^	−0.06 ± 0.03 ^a^	5.82 ± 0.71 ^a^
	3	66.96 ± 1.56 ^bc^	−2.00 ± 0.10 ^d^	1.71 ± 0.19 ^c^
	4	68.53 ± 0.56 ^b^	−2.08 ± 0.10 ^d^	1.60 ± 0.04 ^c^
	5	63.54 ± 0.40 ^d^	−1.65 ± 0.03 ^c^	1.13 ± 0.09 ^c^
65–20	0	94.38 ± 0.74 ^a^	−0.52 ± 0.03 ^b^	3.55 ± 0.20 ^b^
	1	64.25 ± 1.51 ^d^	−2.39 ± 0.46 ^de^	1.62 ± 0.07 ^e^
	2	67.10 ± 0.79 ^c^	0.65 ± 0.25 ^a^	5.24 ± 0.22 ^a^
	3	74.37 ± 1.04 ^b^	−2.59 ± 0.27 ^e^	2.42 ± 0.17 ^c^
	4	65.18 ± 0.90 ^d^	−2.01 ± 0.13 ^cd^	1.67 ± 0.09 ^e^
	5	68.51 ± 0.78 ^c^	−1.91 ± 0.04 ^c^	2.02 ± 0.07 ^d^
65–25	0	95.41 ± 0.91 ^a^	−0.03 ± 0.05 ^a^	3.77 ± 0.23 ^a^
	1	67.39 ± 3.95 ^bc^	−2.19 ± 0.17 ^c^	2.48 ± 0.27 ^b^
	2	64.94 ± 0.89 ^bc^	−0.21 ± 0.03 ^a^	3.78 ± 0.28 ^a^
	3	68.29 ± 1.14 ^b^	−2.60 ± 0.36 ^d^	2.20 ± 0.23 ^b^
	4	64.25 ± 1.22 ^c^	−1.75 ± 0.10 ^b^	1.25 ± 0.15 ^c^
	5	66.67 ± 0.69 ^bc^	−1.96 ± 0.05 ^bc^	2.18 ± 0.18 ^b^
65–30	0	92.72 ± 0.47 ^a^	−0.25 ± 0.02 ^a^	2.57 ± 0.11 ^b^
	1	66.67 ± 2.50 ^c^	−2.04 ± 0.11 ^d^	1.28 ± 0.13 ^e^
	2	64.79 ± 0.76 ^c^	−0.07 ± 0.03 ^a^	3.59 ± 0.04 ^a^
	3	71.51 ± 0.51 ^b^	−2.32 ± 0.26 ^e^	2.75 ± 0.16 ^b^
	4	64.88 ± 1.78 ^c^	−1.80 ± 0.14 ^c^	1.53 ± 0.08 ^d^
	5	64.70 ± 0.62 ^c^	−0.90 ± 0.03 ^b^	2.34 ± 0.11 ^c^
65–35	0	95.95 ± 0.19 ^a^	−0.35 ± 0.01 ^a^	3.57 ± 0.07 ^b^
	1	64.40 ± 1.61 ^d^	−2.24 ± 025 ^b^	1.27 ± 0.06 ^f^
	2	59.47 ± 1.47 ^e^	−0.24 ± 0.09 ^a^	5.11 ± 0.45 ^a^
	3	70.82 ± 1.25 ^b^	−2.24 ± 0.07 ^b^	1.87 ± 0.12 ^e^
	4	67.27 ± 1.56 ^c^	−2.31 ± 0.22 ^b^	2.72 ± 0.27 ^c^
	5	66.54 ± 1.16 ^cd^	−2.17 ± 0.20 ^b^	2.30 ± 0.04 ^d^
65–40	0	94.76 ± 1.68 ^a^	−0.10 ± 0.07 ^b^	4.30 ± 0.53 ^b^
	1	66.85 ± 2.24 ^bc^	−1.72 ± 0.05 ^d^	1.12 ± 0.03 ^d^
	2	64.36 ± 1.03 ^c^	0.87 ± 0.08 ^a^	4.89 ± 0.26 ^a^
	3	64.71 ± 1.17 ^c^	−1.85 ± 0.10 ^e^	0.96 ± 0.28 ^d^
	4	64.37 ± 1.01 ^c^	−1.55 ± 0.08 ^c^	1.67 ± 0.04 ^c^
	5	68.39 ± 0.78 ^b^	−1.91 ± 0.04 ^e^	1.90 ± 0.03 ^c^
**Pasteurization** **conditions** **(°C—s)**	**Storage** **time (d)**	**L***	**a***	**b***
70–15	0	99.61 ± 0.22 ^a^	−0.80 ± 0.01 ^b^	5.48 ± 0.05 ^a^
	1	71.01 ± 1.27 ^b^	−2.16 ± 0.07 ^d^	1.83 ± 0.12 ^d^
	2	65.66 ± 0.59 ^d^	0.19 ± 0.35 ^a^	2.68 ± 0.09 ^c^
	3	72.49 ± 1.47 ^b^	−2.47 ± 0.07 ^d^	2.65 ± 0.05 ^c^
	4	63.32 ± 1.36 ^e^	−1.73 ± 0.09 ^c^	1.24 ± 0.11 ^e^
	5	68.91 ± 1.23 ^c^	−2.39 ± 0.46 ^d^	3.15 ± 0.08 ^b^
70–30	0	91.41 ± 0.35 ^a^	−0.38 ± 0.02 ^b^	3.76 ± 0.09 ^a^
	1	72.35 ± 1.10 ^b^	−2.24 ± 0.07 ^de^	2.39 ± 0.03 ^b^
	2	67.56 ± 0.76 ^d^	0.87 ± 0.08 ^a^	3.53 ± 0.26 ^a^
	3	69.65 ± 1.27 ^c^	−1.97 ± 0.15 ^c^	1.27 ± 0.22 ^d^
	4	69.36 ± 0.29 ^c^	−2.37 ± 0.21 ^e^	1.91 ± 0.13 ^c^
	5	65.22 ± 1.15 ^e^	−2.06 ± 0.07 ^cd^	1.91 ± 0.04 ^c^
75–15	0	98.82 ± 2.90 ^a^	−0.43 ± 0.09 ^a^	5.29 ± 0.26 ^a^
	1	69.77 ± 0.66 ^b^	−2.32 ± 0.28 ^c^	2.18 ± 0.09 ^c^
	2	60.68 ± 1.52 ^d^	−0.24 ± 0.07 ^a^	2.12 ± 0.10 ^c^
	3	68.62 ± 0.83 ^b^	−2.15 ± 0.08 ^c^	1.99 ± 0.07 ^c^
	4	69.52 ± 0.96 ^b^	−2.28 ± 0.08 ^c^	2.98 ± 0.11 ^b^
	5	64.51 ± 0.42 ^c^	−1.60 ± 0.01 ^b^	1.35 ± 0.07 ^d^
75–30	0	98.67 ± 0.45 ^a^	−0.31 ± 0.04 ^b^	4.84 ± 0.10 ^b^
	1	69.57 ± 0.32 ^b^	−2.26 ± 0.05 ^e^	2.65 ± 0.08 ^c^
	2	60.60 ± 2.10 ^d^	−0.06 ± 0.02 ^a^	5.82 ± 0.28 ^a^
	3	63.63 ± 0.75 ^c^	−1.88 ± 0.10 ^d^	0.73 ± 0.14 ^f^
	4	68.17 ± 1.17 ^b^	−2.15 ± 0.07 ^e^	2.33 ± 0.16 ^d^
	5	65.32 ± 1.01 ^c^	−1.60 ± 0.07 ^c^	1.82 ± 0.03 ^e^
80–15	0	92.36 ± 0.54 ^a^	−0.65 ± 0.02 ^b^	4.71 ± 0.09 ^b^
	1	72.42 ± 0.79 ^b^	−2.10 ± 0.06 ^cd^	1.53 ± 0.05 ^d^
	2	61.03 ± 1.95 ^d^	−0.06 ± 0.02 ^a^	5.82 ± 0.28 ^a^
	3	74.01 ± 1.76 ^b^	−2.28 ± 0.27 ^d^	2.29 ± 0.10 ^c^
	4	69.45 ± 1.27 ^c^	−2.17 ± 0.16 ^d^	2.46 ± 0.11 ^c^
	5	61.08 ± 0.17 ^d^	−1.90 ± 0.03 ^c^	2.40 ± 0.03 ^c^
80–30	0	92.53 ± 0.29 ^a^	−0.29 ± 0.02 ^a^	2.86 ± 0.06 ^b^
	1	71.77 ± 0.52 ^b^	−2.36 ± 0.14 ^d^	2.15 ± 0.08 ^c^
	2	61.33 ± 1.00 ^d^	−0.38 ± 0.47 ^a^	4.83 ± 0.64 ^a^
	3	64.69 ± 0.64 ^c^	−1.83 ± 0.12 ^bc^	1.16 ± 0.09 ^d^
	4	65.97 ± 0.88 ^c^	−2.14 ± 0.14 ^cd^	1.69 ± 0.10 ^c^
	5	65.71 ± 1.25 ^c^	−1.69 ± 0.19 ^b^	1.74 ± 0.08 ^c^
85–15	0	96.77 ± 0.41 ^a^	−0.68 ± 0.09 ^b^	5.47 ± 0.62 ^a^
	1	68.35 ± 0.93 ^b^	−2.34 ± 0.13 ^d^	1.46 ± 0.10 ^d^
	2	66.05 ± 0.76 ^c^	0.49 ± 0.07 ^a^	3.54 ± 0.47 ^b^
	3	66.43 ± 0.84 ^c^	−2.15 ± 0.28 ^d^	1.49 ± 0.04 ^d^
	4	67.94 ± 0.58 ^b^	−2.32 ± 0.27 ^d^	2.24 ± 0.07 ^c^
	5	62.47 ± 0.52 ^d^	−1.90 ± 0.03 ^c^	2.43 ± 0.07 ^c^
85–30	0	99.20 ± 0.17 ^a^	−0.30 ± 0.02 ^a^	4.05 ± 0.08 ^b^
	1	74.19 ± 1.08 ^b^	−2.58 ± 0.08 ^d^	4.07 ± 0.39 ^b^
	2	62.84 ± 2.42 ^e^	−0.47 ± 0.12 ^a^	4.95 ± 0.62 ^a^
	3	68.79 ± 0.96 ^c^	−2.06 ± 0.28 ^bc^	1.90 ± 0.05 ^c^
	4	66.24 ± 1.07 ^d^	−2.18 ± 0.05 ^c^	2.04 ± 0.14 ^c^
	5	65.07 ± 0.13 ^d^	−1.90 ± 0.03 ^b^	2.43 ± 0.07 ^c^
90–15	0	102.35 ± 0.70 ^a^	−0.83 ± 0.03 ^b^	5.65 ± 0.24 ^a^
	1	62.68 ± 2.08 ^c^	−1.84 ± 0.08 ^c^	1.02 ± 0.20 ^e^
	2	58.84 ± 1.49 ^d^	−0.29 ± 0.02 ^a^	3.33 ± 0.15 ^b^
	3	66.80 ± 1.64 ^b^	−2.34 ± 0.29 ^d^	2.20 ± 0.15 ^c^
	4	61.17 ± 2.26 ^cd^	−1.86 ± 0.06 ^c^	1.48 ± 0.07 ^d^
	5	68.01 ± 1.59 ^b^	−1.72 ± 0.02 ^c^	1.62 ± 0.03 ^d^
90–30	0	95.20 ± 0.76 ^a^	−0.18 ± 0.04 ^b^	4.01 ± 0.54 ^b^
	1	75.78 ± 0.37 ^b^	−2.61 ± 0.04 ^f^	3.42 ± 0.48 ^b^
	2	68.96 ± 0.44 ^c^	−0.32 ± 0.04 ^c^	5.99 ± 0.82 ^a^
	3	69.54 ± 0.71 ^c^	−2.32 ± 0.08 ^e^	2.21 ± 0.22 ^c^
	4	59.10 ± 2.46 ^d^	−1.82 ± 0.09 ^d^	1.02 ± 0.09 ^d^
	5	59.99 ± 3.97 ^d^	1.66 ± 0.12 ^a^	2.05 ± 0.11 ^c^

L*, lightness; a*, greenness/redness; b*, blueness/yellowness; data represent mean values ± SDs. Different lowercase letters in a column of each stage indicate significant differences (*p* < 0.05).

## References

[1] Nikmaram N., Keener K.M. (2022). The effects of cold plasma technology on physical, nutritional, and sensory properties of milk and milk products. Sci. Direct.

[2] Wu S.Y., Wu S., Han H.J., Kong F.H., Guan B.Y., Zhang X.T., Cao X.Y., Kang S.M., Tao D.B., Yue X.Q. (2018). Comparison of free amino acids in human and cow’s milk at different stages of lactation. Food Sci..

[3] Saini R.K., Keum Y.S. (2018). Omega-3 and omega-6 polyunsaturated fatty acids: Dietary sources, metabolism, and significance. Life Sci..

[4] Liang X., Liu L., Zhang S.W., Sun Q., Lv J.P. (2013). Analysis of Physico-chemical Properties of Raw Milk of Cattle, Buffalo and Yak. Food Sci..

[5] Catanzaro R., Sciuto M., Marotta F. (2021). Lactose intolerance: An update on its pathogenesis, diagnosis, and treatment. Nutr. Res..

[6] Storhaug C.L., Fosse S.K., Fadnes L.T. (2017). Country, regional, and global estimates for lactose malabsorption in adults: A systematic review and meta-analysis. Lancet Gastroenterol. Hepatol..

[7] Albuquerque T.D., Sousa M.D., Silva N. (2021). Beta-Galactosidase from Kluyveromyces lactis: Characterization, production, immobilization and applications. Int. J. Biol. Macromol..

[8] Domg Y.N., Chen W., Zhang H., Gu H.Y., Liu Y., Chen H.Q. (2023). Cumulative effect of mutations at conserved sites of GH42 family on the catalytic activity of *β*-galactosidase BgaB from Geobacillus stearothermophilus. Microbiol. China.

[9] Voisin M.R., Borici-Mazi R. (2016). Anaphylaxis to supplemental oral lactase enzyme. Allergy Asthma Clin. Immunol..

[10] Kocabaş D.S., Lyne J., Ustunol Z. (2022). Hydrolytic enzymes in the dairy industry: Applications, market and future perspectives. Trends Food Sci. Technol..

[11] Singh P., Rao P.S., Sharma V. (2021). Physico-chemical aspects of lactose hydrolysed milk system along with detection and mitigation of maillard reaction products. Trends Food Sci. Technol..

[12] Wang F.N., Zhang Y.D., Zheng N., Wang J.Q. (2021). Research progress on the determination of furosine in milk and dairy products. J. Food Saf. Qual..

[13] Huang Y.S., Lu J.N., Li M.Y., Li C., Sheng M.Y., Xie M.Y. (2023). Effect of Frying Conditions on Acrylamide and 5-Hydroxymethylfurfural Formation in French fries. Food Sci..

[14] Niu L.B., Guo H.L., Huang A.X. (2009). Study of inhibiting effect on 5-HMF in low-lactose milk by compounds of inhibit browning. China Dairy Ind..

[15] Li W., Zhang X.M., Lu Y., Sheng G.Q., Lv J.P., Nan Q.X., Chen L.J. (2004). Browning inhibition techniques for low lactose milks. China Dairy Ind..

[16] Wei M., Chen Y.K., Song X.Y., Luo C.H., Pan X., Huang L. (2018). Analysis of aroma components in proline Maillard reaction by solid phase microextraction. Food Res. Dev..

[17] Zheng X.J., Lin S.L., Nie X.H. (2015). Effect of temperature on spectral characteristics and volatile compounds of Maillard reaction products from xylose-chicken bone hydrolysate system. Food Ferment. Ind..

[18] Liu Y., Jiang W., Yang H.C., Xiang X.W., Hu S.W., Li S.J. (2018). Variables, characterization and preliminary application of Cadaverine/Glucose Maillard reaction. J. Chin. Inst. Food Sci. Technol..

[19] Wang X.D., Kong Y.Z., Zhang Y.L., Ariunjargal T., Munkhjargal B., Wu H.X., Dong A.L.D.E. (2022). Mechanism of sterilization technology and its application in food field. China Brew..

[20] Lambros S., Alexandra M., Ekaterini M. (2014). Assessment of heat treatment of various types of milk. Food Chem..

[21] Zhang W., Poojary M.M.P., Rauh V. (2019). Quantitation of alpha-dicarbonyls and advanced glycation endproducts in conventional and Lactose-Hydrolyzed ultrahigh temperature milk during 1 Year of Storage. J. Agric. Food Chem..

[22] Liu J.B., Wang Z.Q., Yu Y.D., Zhang T., Liu B.Q. (2021). Optimization of preparation technology of soybean meal peptides by response surface methodology. J. Chin. Inst. Food Sci. Technol..

[23] Li R.M., Zhang H.X., Pan S.K., Ye J.J. (2023). Process optimization and functional properties of peony seeds protein. Sci. Technol. Food Ind..

[24] Shan Q.Y., Shen Y.M., Zhang M.X., Tang J.C., Chen Y.J., Bao J.Q. (2022). Optimization of ultrasonic-compound enzyme hydrolysis method for extracting Chinese softshell turtle oil by orthogonal experimen. Sci. Technol. Food Ind..

[25] Li S.N., Tang S.H., Hu Y., Mao M.L., Liu Y. (2017). Effect of enzymatic hydrolysis of lactose combined with heat treatments on Maillard Reaction of milk. Food Sci..

[26] Giannetti V., Mariani M.B., Colicchia S. (2021). Furosine as marker of quality in dried durum wheat pasta: Impact of heat treatment on food quality and security. Food Control.

[27] Wei X.X., Xiao G.N., Xiong W., Zhao G.S., Chen B., Gong J.Y. (2021). Effects of low heat treatments on furosine in milk simulation system. J. Chin. Inst. Food Sci. Technol..

[28] Hao X.Y., Chen M.X., Liu H.M., Zang C.J., Zheng N., Wang J.Q. (2022). Effects of storage and transportation conditions on the furosine content in UHT milk. China Dairy Ind..

[29] Sunds A.V., Rauh V.M., SøRensen J. (2017). Maillard reaction progress in UHT milk during storage at different temperature levels and cycles. Int. Dairy J..

[30] Troise A.D., Buonanno M., Fiore A. (2016). Evolution of protein bound Maillard reaction end-products and free Amadori compounds in low lactose milk in presence of fructosamine oxidase I. Food Chem..

[31] Bai Y.S., Yang X.J., Li M., Xu Y.Y. (2018). Study on furosine in sterilized milk products under different production processes and storage conditions. J. Food Saf. Qual..

[32] Ferrer E., Amparo A., Rosaura F. (2005). High-performance liquid chromatographic determination of furfural compounds in infant formulas during full shelf-life. Food Chem..

[33] Liu X.Y., Chen X.L., Lin T., Wang L., Wang L.X., Li M.X., Shao J.L. (2022). Effects of Roasting Degree on Formation of 5-Hydroxymethylfurfural contents in coffea arabic. J. Chin. Inst. Food Sci. Technol..

[34] Małgorzata C., Renata P.B.B.K. (2020). Evaluation of 5-hydroxymethylfurfural content in market milk products. Food Addit. Contam. Part A.

[35] Gao M., Ge W.P., Zhang X.Y., Cui L.L., Qin L.H. (2012). Study on the quantities change of the 5-Hydroxymethylfurfural in milk under the different heat treatment. Food Ind..

[36] Bi R.X., Lu J., Zhang S.W., Pang X.Y., Hu C., Yu J.H., Lv J.P. (2021). Changes of Maillard reaction by-products in brown yogurt under differentd storage conditions. Food Ferment. Ind..

[37] Jia H.X., Su M.Y., Chen W.L., Liu H.A. (2022). Research progress on Maillard Reaction indicators of infant formula Milk powde. Food Ind..

[38] Zhao Y., Li L.Q., Niu P.F. (2020). Optimization of reverse-phase high-performance liquid chromatography(RP-HPLC) method for determination of 5hydroxymethylfurfural in milk. Jiangsu J. Agric. Sci..

[39] Cai L., Li D., Dong Z. (2016). Change regularity of the characteristics of Maillard reaction products derived from xylose and Chinese shrimp waste hydrolysates. LWT—Food Sci. Technol..

[40] Meng Y.C., He S.S., Li Y.H., Chen J., Shen L.M. (2015). Study on the Maillard Reaction in milk under different heating conditions. Mod. Food Sci. Technol..

[41] Yang X.Q., Liu X.M., Zhou P. (2012). Application of front-face fluorescence spectroscopy for determination of Maillard reaction in milk. Sci. Technol. Food Ind..

[42] Li Y., Fang L., Luo C. (2009). Functional properties of the Maillard reaction products of rice protein with sugar. Food Chem..

[43] Tan J.N., Yao Y., Wu N., Xu M.S., Zhao Y., Liu H.P., Tu Y.G. (2022). Effect of temperature on color, physicochemical characteristics and antioxidant activities of ovalbumin-glucose system under alkaline condition. Food Sci..

[44] Yi S.N., Lu J., Pang X.Y., Hao L.Y., Zhang S.W., Lv J.P. (2021). Effect of heat treatment on Maillard Reaction degree and volatile componentsof milk. Food Sci..

[45] Yokogawa T., Yamazaki C., Hara M. (2023). Effect of Maillard reaction on the quality of clarified butter, ghee. J. Nat. Med..

[46] Chiku K. (2023). Research on Structural transformation of oligosaccharides under heat treatment. Bull. Appl. Glycosci..

[47] Shen Y., Dong Q., Yu Z. (2023). The effects of free radicals and metal ion on milk protein β-lactoglobulin (BLG) glyco-oxidation. Int. J. Food Sci. Technol..

[48] Poojary M.M., Lund M.N. (2022). Chemical stability of proteins in foods: Oxidation and the Maillard Reaction. Annu. Rev. Food Sci. Technol..

[49] Wang Z.H., Liu D.Y., Zheng J.C., Xie Y.T., Han J.C., Wang Y.N. (2021). Preparation and emulsifying properties of Maillard Reaction products of soybean protein isolate under high hydrostatic pressure. Sci. Technol. Food Ind..

[50] Chen L., Haoran X., Zhiyan C. (2014). Comparative studies on the physicochemical properties of peanut protein isolate–polysaccharide conjugates prepared by ultrasonic treatment or classical heating. Food Res. Int..

[51] Zhang Y.Y., Huang X., Yan D.Y., Zhao W.J., Hao J.X., Liu J.G. (2022). Preparation and properties of sodium caseinate-oat β-glucan conjugatesobtained by the Maillard reaction. Fine Chem..

[52] Li Z.C., Xu M.F., Xiang M.X., Cheng X.F. (2013). Research on the Structural Differences of the Casein from Milk by Pasteurization and Ultrahigh Temperature Sterilization. J. South China Agric. Univ..

[53] Wang L., Lv J.L., Wang S. (2014). Analysis of reducing sugar in yogurt by pre-column derivatization HPLC. China Dairy Ind..

[54] Song H.M., Lu J., Lv J.P., Zhao W.L., Li D.H., Zhu Y.M. (2016). Evaluation of the degree of heating and flavor change of milk based on electronic nose and electronic tongue. China Dairy Ind..

[55] Yazdi S.R., Corredig M. (2012). Heating of milk alters the binding of curcumin to casein micelles. A fluorescence spectroscopy study. Food Chem..

